# Siderophore-Mediated Mobilization of Manganese Limits Iron Solubility in Mixed Mineral Systems

**DOI:** 10.1021/acsearthspacechem.2c00271

**Published:** 2023-03-09

**Authors:** Kyounglim Kang, Jasquelin Peña

**Affiliations:** † Department of Civil and Environmental Engineering, University of California, Davis, California 95616, United States; ‡ Energy Geosciences Division, Lawrence Berkeley National Laboratory, Berkeley, California 94720, United States

**Keywords:** aqueous Mn(III), δ-MnO_2_, manganite, lepidocrocite, 2-line ferrihydrite, iron acquisition, siderophore, DFOB

## Abstract

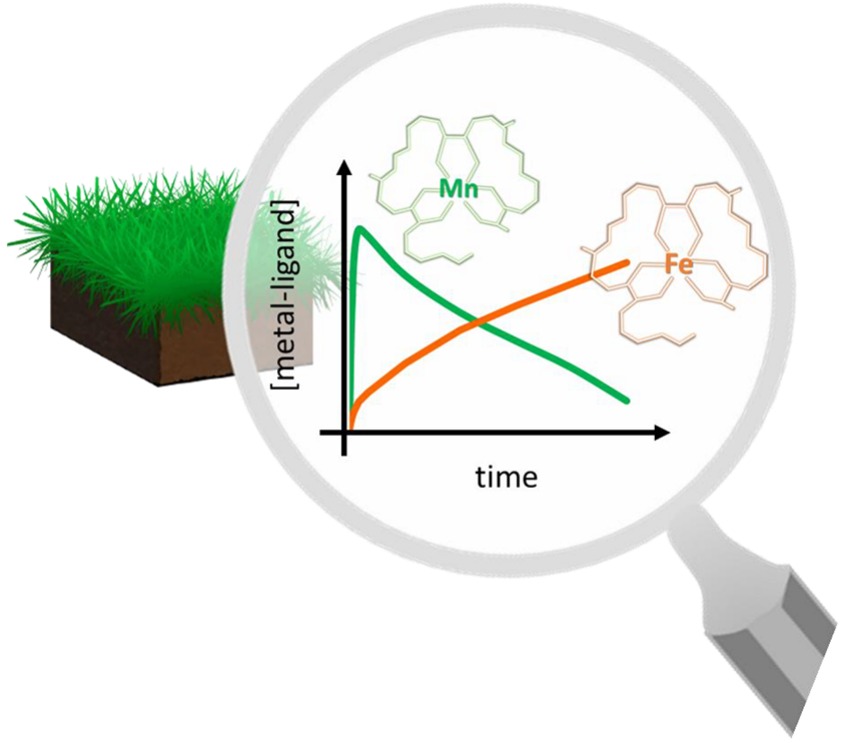

Recent laboratory and field studies show the need to consider the formation of aqueous Mn(III)-siderophore complexes in manganese (Mn) and iron (Fe) geochemical cycling, a shift from the historical view that aqueous Mn(III) species are unstable and thus unimportant. In this study, we quantified Mn and Fe mobilization by desferrioxamine B (DFOB), a terrestrial bacterial siderophore, in single (Mn or Fe) and mixed (Mn and Fe) mineral systems. We selected manganite (γ-MnOOH), δ-MnO_2_, lepidocrocite (γ-FeOOH), and 2-line ferrihydrite (Fe_2_O_3_·0.5H_2_O) as relevant mineral phases. We found that DFOB mobilized Mn(III) as Mn(III)-DFOB complexes to varying extents from both Mn(III,IV) oxyhydroxides but reduction of Mn(IV) to Mn(III) was required for the mobilization of Mn(III) from δ-MnO_2_. The initial rates of Mn(III)-DFOB mobilization from manganite and δ-MnO_2_ were not affected by the presence of lepidocrocite but decreased by a factor of 5 and 10 for manganite and δ-MnO_2_, respectively, in the presence of 2-line ferrihydrite. Additionally, the decomposition of Mn(III)-DFOB complexes through Mn-for-Fe ligand exchange and/or ligand oxidation led to Mn(II) mobilization and Mn(III) precipitation in the mixed-mineral systems (∼10% (mol Mn/mol Fe)). As a result, the concentration of Fe(III) mobilized as Fe(III)-DFOB decreased by up to 50% and 80% in the presence of manganite and δ-MnO_2_, respectively, compared to the single mineral systems. Our results demonstrate that siderophores, through their complexation of Mn(III), reduction of Mn(III,IV), and mobilization of Mn(II), can redistribute Mn to other soil minerals and limit the bioavailability of Fe in natural systems.

## 1 Introduction

Iron (Fe) and manganese (Mn) are the first and second most abundant redox-active elements in the Earth’s crust, respectively.^1,2^ Both trace metals are essential nutrients for all living organisms and their redox cycles impact numerous environmental processes, including nutrient cycling,^3,4^ soil carbon stabilization,^5,6^ and contaminant (im)mobilization.^7−9^ In soils, Fe and Mn often co-occur as various Fe(III) and Mn(III,IV) oxyhydroxide phases.^10^ Due to their ubiquitous nature, large surface area, and high redox activity, these metal oxides inevitably interact through electron transfer between Fe(II) and Mn(III,IV) oxyhydroxides^11^ or surface catalyzed Mn(II) oxidation by Fe(III) oxyhydroxides.^12^ Organic acids, which can promote ligand-promoted and reductive dissolution of Fe(III) and Mn(III,IV) oxyhydroxide phases, can provide another important link between the Fe and Mn cycles.^13^

Laboratory studies have shown that Mn(III) species form through four processes: (1) abiotic and biotic oxidation of Mn(II) to Mn(III),^14^ (2) comproportionation of Mn(II) and Mn(IV) at the surface of Mn(IV) oxides,^15^ (3) photo^16^/chemical^17−20^ reduction of Mn(IV) oxyhydroxides, and (4) ligand-promoted dissolution of Mn(III,IV) oxyhydroxides.^21−23^ Due to the rapid disproportionation of free Mn(III) ions to Mn(II) and Mn(IV)O_2_, these reactions are restricted to conditions where the solution pH is extremely low^24^ or where Mn(III) binding ligands are present.^25^ Until recently, the prevalence and environmental significance of dissolved Mn(III) species had been ignored due to their low solubility and redox instability.^26−28^ However, substantial amounts of aqueous Mn(III) complexed by organic and inorganic ligands have been found in natural systems, including in soil,^29,30^ sediment,^31,32^ estuarine,^33−35^ and marine^36,37^ environments.

The most common class of Mn(III) binding ligands are those with a high affinity for Fe, known as siderophores.^38,39^ Plants and microorganisms exude siderophores in response to Fe deficiency, which typically occurs at circumneutral pH and oxic conditions where Fe solubility is low.^40,41^ The hydroxamate,^14,39^ (amino)carboxylate,^37^ catechol,^42^ or phosphate^43^ functional groups in siderophores form stable aqueous complexes with Fe(III). Though generally considered specific for Fe(III), nearly all siderophores have a similar or higher affinity for Mn(III).^44^ Duckworth and Sposito^14^ showed that the bacterial siderophore, desferrioxamine B (DFOB), has comparable affinities for Mn(III) and Fe(III) and can mobilize Mn from Mn(III,IV) oxyhydroxide minerals as Mn(III)-DFOB and Mn(II).^22,45^ Any siderophore-mediated mobilization of Mn(III) may simultaneously lower the pool of ligands available for Fe chelation and promote the redistribution of Mn. Additionally, as shown by the redox ladder in Figure S1, the high reduction potential of Mn(III,IV) oxyhydroxides may lead to ligand oxidation and thus inhibit siderophore-assisted Fe mobilization.

Despite the multiple pathways through which siderophores may impact Fe and Mn cycling, prior studies have focused only on siderophore-promoted mobilization of metals from single mineral systems. The extents of ligand competition, decomposition of the ligand and/or metal–ligand complexes, and steady-state concentrations of the metal complexes in the presence of multiple redox-active minerals are therefore unknown.^40,46−48^ In this study, we investigated the kinetics of Mn(III)-ligand, Mn(II), and Fe(III)-ligand mobilization by DFOB from single (Mn or Fe) and mixed (Mn and Fe) mineral systems composed by manganite (γ-MnOOH), δ-MnO_2_, lepidocrocite (γ-FeOOH), and/or 2-line ferrihydrite (Fe_2_O_3_·0.5H_2_O).

Knowing that the mobilization kinetics and the stability of Mn and Fe species depend on pH, mineral type, and mineral combination, we developed two hypotheses. First, we hypothesized that DFOB can mobilize Fe as Fe(III)-DFOB and Mn as both Mn(III)-DFOB and Mn(II), with Mn(II) resulting from ligand-assisted reduction of Mn(IV) or Mn(III). Second, we hypothesized that Mn(III)-DFOB and Mn(II) may readsorb onto Fe(III) oxyhydroxides, leading to Mn precipitation. To test these hypotheses, we conducted dissolution and adsorption experiments where DFOB was added to suspensions of Mn(III,IV) oxyhydroxides and/or Fe(III) oxyhydroxides at circumneutral pH. Additionally, to determine the oxidation state and local bonding environment of any Mn associated with the Fe(III) minerals, we collected Mn–K edge X-ray absorption (XA) spectra from (i) Fe(III) oxyhydroxides reacted with Mn(III)-DFOB and (ii) Fe(III) oxyhydroxides reacted with Mn(III,IV) oxyhydroxides and DFOB. Based on metal mobilization kinetics and dissolved and solid-phase speciation analyses in model laboratory systems, we elucidated the pathways through which siderophores can mobilize Fe and Mn in natural environments where these metals co-occur.

## 2 Materials and Methods

### 2.1 Materials

All chemicals were obtained from commercial sources (Table S1) and used as received. Ultrapure water (resistivity >18.2 MΩ·cm, TOC < 2 ppb, Milli-Q, Millipore) was used to prepare all solutions and suspensions.

#### 2.1.1 Mineral Preparation

The four minerals used in this study were chemically synthesized using established protocols. Manganite and δ-MnO_2_ were synthesized according to Hens et al.^49^ and Marafatto et al.,^16^ respectively. Lepidocrocite and 2-line ferrihydrite were synthesized according to Schwertmann and Cornell (2008),^50^ as described in detail in Mørup et al.^51^ and Schwertmann et al.,^52^ respectively. Lepidocrocite and 2-line ferrihydrite were stored as aqueous suspensions and used within 1 month of being synthesized; δ-MnO_2_ was also stored as an aqueous suspension. Manganite was stored as a dried powder in the freezer. Prior to use, the manganite powder was suspended in Mili-Q water and sonicated for 5 min to achieve particle dispersion.

#### 2.1.2 Mineral Characterization

Mineral purity and crystallinity were determined by powder X-ray diffraction (XRD) using a Bruker AXS D8 Advance powder diffractometer (Cu Kα radiation, l = 1.5406 Å) at 40 kV and 40 mA. Crystallographic phase and lattice constants were confirmed using Jade MDI software (Figure S2). Mineral specific surface area (SSA) was determined by the Brunauer–Emmet–Teller (BET) method with nitrogen adsorption using a Gemini VII Surface Area Analyzer (Micromeritics Instrument Corp., Norcross, GA) (Table S2). Finally, the average manganese oxidation numbers (AMON, Table S2) of manganite and δ-MnO_2_ were determined by a three-step titration^16,53,54^ by using a Metrohm 906 Titrando titrator equipped with a Pt potentiometric electrode. Briefly, a reference solution of Mohr’s salt (1 mM in 0.1% sulfuric acid) was titrated with KMnO_4_ (1 mM) to count the total moles of Fe(II) (*V*_0_: volume of KMnO_4_ solution added to the reference solution). Second, the Mn(III,IV) oxyhydroxides were dissolved in a Mohr’s salt solution of the same mass as the reference solution. This test solution was titrated with KMnO_4_ to quantify the number of moles of Fe(II) oxidized during the reduction of Mn(III,IV) oxyhydroxide minerals (*V*_1_: amount of KMnO_4_ solution added to the sample solution). Third, excess pyrophosphate (PP, ∼225 mM) was added to the test solution and the pH was adjusted to 6.5 before titration with KMnO_4_ to oxidize Mn(II) to Mn(III) and therefore determine the total amount of Mn in the sample (*V*_2_: volume of KMnO_4_ solution added to the PP-containing test solution). The AMON value was then calculated as 2 × [(2 + 5(*V*_0_ – *V*_1_)/(4 *V*_2_ – *V*_1_)].^54^

### 2.2 Chemical Analysis

#### 2.2.1 Dissolved Species

Total dissolved Mn and Fe concentrations were measured by inductively coupled plasma mass spectrometry (ICP-MS, Agilent-7900). In this study, the total dissolved Fe includes only Fe(III)-DFOB as determined from the agreement between ICP-MS and UV–vis spectrophotometry measurements of Fe-DFOB in control experiments while the total dissolved Mn includes Mn(II) and Mn(III)-DFOB. The dissolved Mn(II) was calculated as the difference between total dissolved Mn and Mn(III)-DFOB, which was quantified by UV–vis spectrometry as described below. The instrument was equipped with a quartz spray chamber, a microMist concentric gas nebulizer, and nickel sampler and skimmer cones. The instrument was operated using a 1.0 L min^–1^ flow rate of argon carrier gas in helium (He) mode; the He flow rate was maintained at 4.5 mL min^–1^ to minimize polyatomic interferences. The limit of quantification, which was calculated as a 3.3× detection limit,^55^ was 0.04 μg L^–1^ (0.67 nM) for Mn and 0.2 μg L^–1^ (4.4 nM) for Fe.

Aqueous concentrations of Fe(III)-DFOB, Mn(III)-DFOB, and Mn(III)-pyrophosphate (hereafter, Fe-DFOB, Mn-DFOB, and Mn-PP) were determined by UV–vis spectrophotometry (UV-2600, SHIMADZU). For Fe-DFOB, standard solutions were prepared by combining Fe(III) and DFOB solutions in a 1:1 ratio. The Fe-DFOB solution has a dark orange coloration with an absorbance maximum at 428 nm (ε_428_ = 2820 M^–1^ cm^–1^) at pH 7.0 and 7.5 (ε_428_ = 2831 M^–1^ cm^–1^; Figure S3a). For Mn-DFOB, standard solutions were prepared by air-oxidation of a Mn(II) solution in the presence of DFOB at pH 9.0. The Mn(II) to DFOB ratio was 1:1.1 in order to ensure that all of the oxidized Mn(II) was complexed with DFOB.^37^ The Mn-DFOB solution has a dark green coloration with an absorbance maximum at 310 nm (ε_310_ = 2230 M^–1^ cm^–1^) at pH 7.0 and 7.5 (Figure S3b). The presence of Mn(II) did not interfere with the absorbance of Mn-DFOB (ε_310_ = 2244 M^–1^ cm^–1^, Figure S3c). However, Fe-DFOB, when present, interfered with Mn-DFOB quantification since both complexes absorb light at 310 nm (Figure S3d). In these samples, absorbance due to Mn-DFOB was calculated by subtracting the absorbance due to Fe-DFOB from the total absorbance. These calculations are described in Text S1, with the relevant absorption coefficients tabulated in Table S3. For Mn-PP, standard solutions were prepared by the addition of a pyrophosphate solution to Mn(III)-acetate particles with a ratio of 20:1. The solutions were stirred vigorously under a N_2(g)_ environment for 24 h and filtered prior to measurement. The Mn-PP solution has a light pink coloration with an absorbance maximum at 257 nm (ε_257_ = 6776 M^–1^ cm^–1^) at pH 8.0 (Figure S3e). The presence of Mn(II) did not interfere with the absorbance of Mn-PP (ε_257_ = 6766 M^–1^ cm^–1^) (Figure S2f).

Finally, the concentration of uncomplexed DFOB was determined by complexation of DFOB with Fe(III), where Fe(III) solution was added in a small excess over the ligand concentration to ensure complete complexation of DFOB by Fe. The excess Fe was allowed to precipitate overnight. The following day, the sample was filtered (0.22 μm polyethersulfone (PES) filter) to remove any Fe(III) precipitates and the filtrate was analyzed by UV–vis spectrophotometry.

#### 2.2.2 Solid-Phase Mn(III)

Pyrophosphate (PP) extractions^56^ were used to determine the concentration of solid-phase Mn(III) in δ-MnO_2_. Briefly, a PP solution was used to extract Mn(III) from the solid and the resulting Mn-PP complex was quantified by UV–vis spectrophotometry. Prior to analysis, the δ-MnO_2_ particles were washed with 0.1 M NaCl three times by centrifugation-resuspension cycles.^57^ The washed particles were resuspended in a PP solution at a 1:20 Mn/PP ratio at pH 6.5 for 24 h in the dark. After 24 h, the suspension was filtered through a 0.22 μm PES filter, and the solution was used for Mn–PP quantitation.

#### 2.2.3 Manganese K-edge X-ray Absorption Spectroscopy

Manganese K-edge XA spectra for a subset of samples (see Section 2.3.3) were collected at 77 K (LN2 cryostat) at the Stanford Synchrotron Radiation Lightsource (BL 4-1) using a Si(220) ϕ = 90 monochromator crystal. The incident beam (1 mm in the vertical dimension) was detuned by 50% at 7000 eV. Monochromator energies were calibrated using a metal foil at 6539 eV (Mn). Manganese K-edge XA spectra were collected in fluorescence mode. Fluorescence yield spectra were measured using a solid-state passivated implanted planar silicon (PIPS) detector or a germanium (Ge) detector equipped with Z-1 filters (i.e., Cr for Mn). Data reduction was completed using standard procedures. Replicate scans (4–5) were averaged to improve the signal-to-noise ratio, and the dead-time was corrected when acquired using the Ge detector. No X-ray-induced changes were observed between replicate scans. X-ray absorption spectra were averaged, background subtracted (*E*_0_ of 6550 for Mn), and normalized to 1 absorption unit using Sixpack.^58^ The extracted extended X-ray fine structure (EXAFS) spectra were weighted by k^3^ and Fourier-transformed (FT) using a Kaiser–Bessel window with a dk value of 3 Å^–1^. Both X-ray near edge structure (XANES) and EXAFS spectra were compared to reference spectra acquired from samples with Mn in known oxidation states. Manganese K-edge XANES and EXAFS spectra were subsequently analyzed by linear combination fitting (LCF) in Athena.^59^

#### 2.2.4 DFOB and Oxidation Products

Liquid chromatography–mass spectrometry (LC–MS) was used to determine the DFOB concentration and to identify any DFOB oxidation products. For LC–MS analysis, the sample was diluted in H_2_O and injected into a Phenomenex Kinetex C18 column (2.1 mm × 100 mm). Two solvents were used (Optima LC–MS grade, Thermo Fisher Scientific, Waltham, MA): H_2_O/0.1% formic acid (A solution) and acetonitrile (B solution). A reverse phase gradient was run over 12 min, with the percentage of the B solution increasing from 5% to 90% at a flow rate of 250 μL min^–1^. The HPLC eluent was monitored for positive and negative ions *via* separate LC runs using a Thermo Scientific Q-Exactive HF mass spectrometer (Bremen, Germany) operated in profile mode. Source parameters were 4.5 kV spray voltage, capillary temperature of 275 **°**C, and sheath gas setting of 15. Spectral data were acquired at a resolution setting of 60,000 fwhm (full width at half maximum) with the lockmass feature enabled, which typically results in a mass accuracy <2 ppm.

### 2.3 Dissolution and Adsorption Experiments

#### 2.3.1 Dissolution of Mn(III/IV)/Fe(III) Oxyhydroxide Minerals

Dissolution experiments were carried out under ambient atmospheric conditions at constant temperature (20 ± 1 °C) in a 0.1 M NaCl background electrolyte. The ionic strength of 0.1 M NaCl was selected to avoid any changes in the reaction progress due to changes in the ionic strength of the solution upon DFOB addition or reaction product accumulation. All experiments were conducted in duplicate. The batch reactors (glass beaker, 100 mL) were wrapped in aluminum foil to prevent potential photo-reduction of Mn(III,IV)/Fe(III) oxyhydroxide minerals or photo-decomposition of the metal–ligand complexes. The reactor contents were mixed continuously with a magnetic stirrer and a Teflon-coated stirring bar. The pH was set by the addition of 10 mM HCl or NaOH and maintained constant (ΔpH = ± 0.05) by using a pH STAT (Metrohm, 906 Titrando). The volumes of NaOH and/or HCl added for pH adjustment (less than 1 mL) were recorded and accounted for when calculating the concentrations of all dissolved species. We avoided the use of organic buffers for pH control because Good’s buffers are recognized to reduce Mn(IV) and create Mn(III) in layer-type Mn oxides.^17,57,60^

To begin an experiment, an aliquot of the mineral stock suspension was added to the background electrolyte at 90% of the final suspension volume. The suspension pH was adjusted to the desired value using 10 mM HCl or NaOH. After 1 h of equilibration, the final volume (50 mL) and suspension concentrations (1 mM Fe and/or Mn, 50 μM DFOB, 0.1 M NaCl) were reached by the addition of DFOB from a stock solution (1 mM DFOB). The initial time (*t* = 0) corresponds to the time of DFOB addition.

Samples were collected periodically over a period of 72 h and filtered through 0.22 μm PES syringe filters. The filtrates (2.5 mL) were collected and split into two aliquots, such that one sample aliquot was acidified with trace metal grade HNO_3_ for analysis of the total dissolved Mn and Fe concentrations and one sample aliquot was measured immediately to determine the Mn-DFOB and Fe-DFOB concentrations as described in Section 2.2.1.

The initial rates of Mn-DFOB, Fe-DFOB, and Mn(II) mobilization were calculated by linear regression of the dissolved Mn-DFOB, Fe-DFOB, and Mn(II) concentration against time for the data points over which the increase of dissolved Mn and Fe was linear. The initial rates (mol kg^–1^ h^–1^) were normalized by the mass of mineral (kg) in the reactor. The rate of DFOB decomposition, instead, was modeled as a first-order process with the rate coefficient calculated by linear regression of the log of the Mn-DFOB concentration against time.

#### 2.3.2 Metal and Ligand Adsorption

Adsorption isotherms were measured for sorption of Mn(II) (0–500 μM Mn(II); pH 7.0 and 7.5), DFOB (0–100 μM; pH 7.0 and 7.5), Mn-DFOB (0–100 μM; pH 7.0), and Fe-DFOB (0–100 μM; pH 7.0) to Mn(III,IV) and Fe(III) oxyhydroxide minerals. Mineral suspensions were prepared as described in Section 2.3.1. Experimental determination of Mn(II) adsorption onto δ-MnO_2_ was not possible due to the fast oxidation of adsorbed Mn(II) even under anoxic conditions^61,62^ (data not shown). All experiments were carried out in duplicate at 20 ± 1 °C, in a 0.1 M NaCl electrolyte and under ambient atmospheric conditions, except Mn(II) adsorption experiments, which were conducted under a N_2_-atmosphere to avoid surface oxidation of Mn(II).

Adsorption experiments were initiated by adding an aliquot of the adsorptive to the mineral suspension (50 mL) contained in a glass beaker (100 mL). Samples were mixed by using a magnetic stirrer and a Teflon-coated stir bar. After a reaction time of 10 min, samples were filtered (0.22 μm PES filter) and the filtrate was analyzed for the dissolved metal and metal–ligand concentrations as described in Section 2.2.1. The *q*_max_ and *K*_D_ values (Table S6) were calculated by fitting a Langmuir model to the experimental data (Figures S8–S11).^63^

#### 2.3.3 Samples for XAS Analysis

Two types of sample were used for X-ray absorption spectroscopy (XAS) analysis (Section 2.2.3): Type 1 samples were collected from experiments where Mn-DFOB was reacted with Fe(III) oxyhydroxides and Type 2 samples were collected from experiments where DFOB was reacted with Fe(III) oxyhydroxides in the presence of Mn(III/IV) oxyhydroxides. These experiments were conducted at pH 7 on a pH STAT. For Type 1 samples, 1 mM Mn-DFOB was reacted with lepidocrocite or 2-line ferrihydrite (10 mM Fe, 0.1 M NaCl) for 10 days. For Type 2 samples, a dialysis membrane device with 1000 kDa molecular weight cutoff (Float-A-Lyzer, Spectrum Laboratory Products) was used to physically separate the Fe and Mn minerals. Mineral suspensions to final concentrations of 10 mM Mn and 10 mM Fe were added in two separate Float-A-Lyzer devices. Subsequently, the devices were submerged in a 100 mL reactor containing 0.1 M NaCl and 1 mM DFOB for 10 days. Parallel experiments for the analysis of dissolved species (Section 2.2.1) were conducted under the same conditions as described in Section 2.3.1 but without the Float-A-Lyzer devices.

After 10 days of reaction, the solids were collected for XAS analysis by filtration onto 0.45 μm filter membranes (25 mm diameter, nitrocellulose filter; Millipore Sigma-Aldrich). The filter membranes were cut into 0.2 × 1.8 cm^2^ rectangles, stacked, and sealed with Kapton tape. A sample mass of about 8.1 mg in a 0.36 cm^2^ area was used in order to avoid self-absorption effects. The sealed samples were attached to an aluminum sample holder and stored at −20 °C until analysis to prevent any changes in the Mn redox state.

## 3 Results

### 3.1 Ligand-Promoted Mn and Fe Mobilization

#### 3.1.1 Mn-DFOB Mobilization from Mn(III,IV) Oxyhydroxides: Effect of Fe(III) Oxyhydroxides

**Figure 1:**
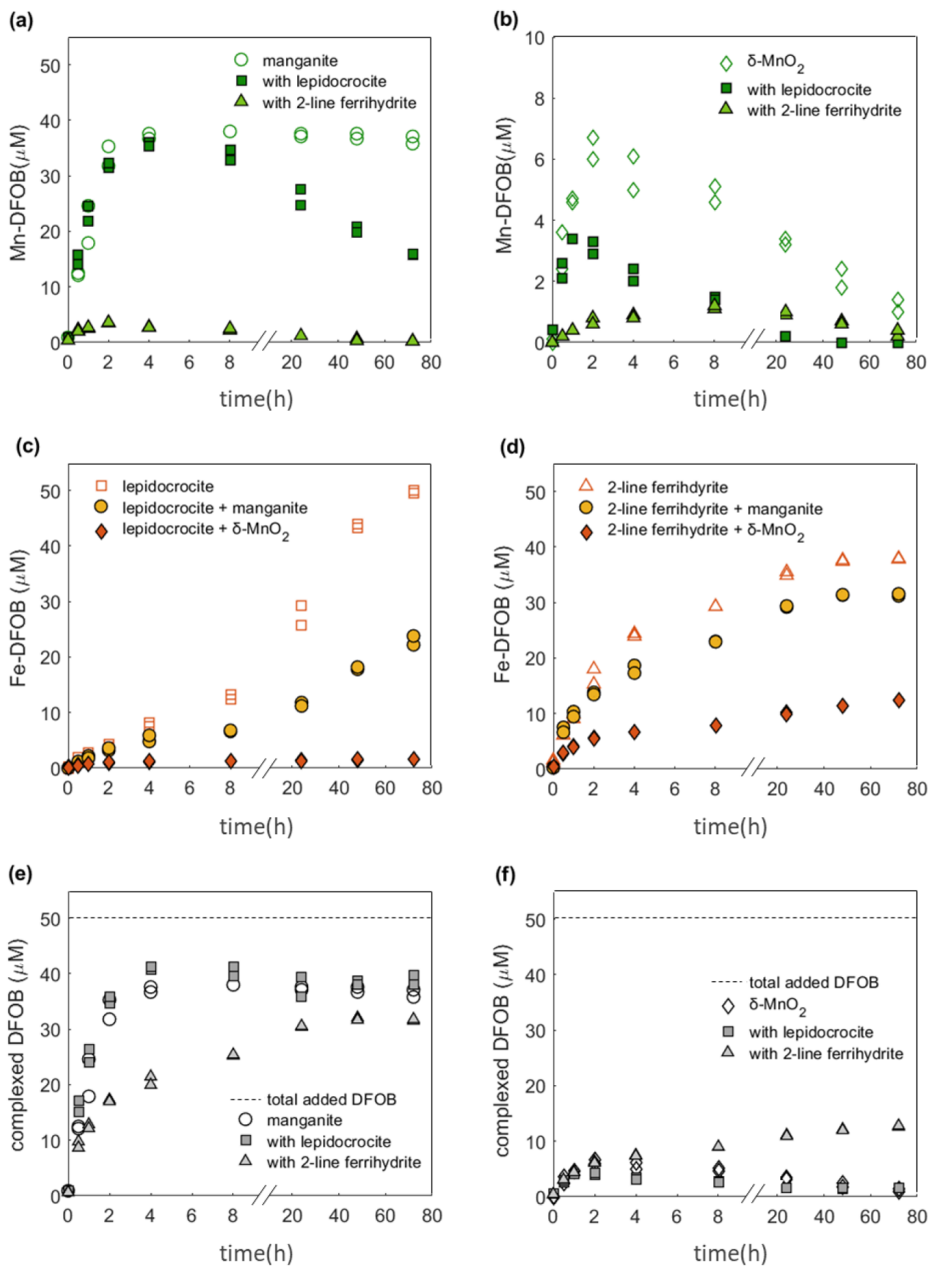
Manganese (Mn) mobilization from (a) manganite and (b) δ-MnO_2_ and iron (Fe) mobilization from (c) lepidocrocite and (d) 2-line ferrihydrite by 50 μM DFOB as a function of time at pH 7.0 under oxic conditions in single and mixed mineral suspensions (1 mM Mn and 1 mM Fe, 0.1 M NaCl). Mobilized Mn(III) and Fe(III) are shown as Mn-DFOB (a and b) and Fe-DFOB (c and d), respectively. Complexed DFOB is shown as Mn-DFOB in Mn single mineral systems and the sum of Mn-DFOB and Fe-DFOB in mixed mineral systems (e and f). A different *y*-axis scale was used for (b).

Figure 1a shows the mobilization of Mn-DFOB from manganite (1 mM Mn, AMON: 3.02) by 50 μM DFOB in the absence and presence of lepidocrocite and 2-line ferrihydrite (1 mM Fe) at pH 7.0. Experiments conducted at pH 7.5 showed marginal differences in reaction rates and extent relative to pH 7.0 and hence are not discussed further (Figure S5a and Table S4). In the absence of the Fe(III) oxyhydroxide minerals, the Mn-DFOB concentration increased linearly during the first 2 h (0.15 mol kg^–1^ h^–1^) and reached a maximum concentration of 38 μM after 4 h. With added lepidocrocite, the initial mobilization rate and maximum concentration of Mn-DFOB were comparable to the manganite-only system (Table S4). With added 2-line ferrihydrite, the initial mobilization rate of Mn-DFOB (0.034 mol kg^–1^ h^–1^) and the maximum concentration of Mn-DFOB (3.5 μM) were suppressed by a factor of 4.4 and 10.9, respectively, compared to the manganite-only system. Furthermore, in comparison to the manganite-only treatment where the maximum Mn-DFOB concentration remained constant from 4 to 72 h, the Mn-DFOB concentration decreased to 15.8 and 0.25 μM in the treatments with lepidocrocite and 2-line ferrihydrite, respectively. Finally, Mn-DFOB decomposition was faster in the presence of 2-line ferrihydrite (0.035 h^–1^) than in the presence of lepidocrocite (0.012 h^–1^) due to more favorable adsorption of Mn-DFOB complexes onto 2-line ferrihydrite than lepidocrocite (Table S4 and Figure S10) and subsequent Mn-for-Fe metal exchange as discussed in Section 4.2.

The mobilization kinetics of Mn-DFOB from δ-MnO_2_ (1 mM Mn, AMON: 4.01) at pH 7.0 are shown in Figure 1b. In the absence of Fe(III) oxyhydroxide minerals, the Mn-DFOB concentration increased linearly over 2 h (0.029 mol kg^–1^ h^–1^) to a maximum concentration of 6.7 μM. However, after 2 h, the Mn-DFOB concentration decreased over time (0.023 h^–1^, Table S4), which indicates that Mn-DFOB is unstable in the presence of δ-MnO_2_. In the presence of the Fe(III) oxyhydroxides, the initial mobilization rates of Mn-DFOB were lower by a factor of 2.4 for the lepidocrocite + δ-MnO_2_ and 9.6 for 2-line ferrihydrite + δ-MnO_2_ treatments (Table S4). Additionally, the maximum mobilized Mn-DFOB concentrations decreased by a factor of 2.0 and 6.4 for the lepidocrocite + δ-MnO_2_ and 2-line ferrihydrite + δ-MnO_2_ treatments, respectively, compared to the δ-MnO_2_-only treatment. The decomposition rate coefficient of Mn-DFOB in the presence of 2-line ferrihydrite (0.020 h^–1^) was smaller than in the presence of lepidocrocite (0.075 h^–1^). After 72 h of reaction, the Mn-DFOB concentrations were low but measurable: 1.2, 0.02, and 0.3 μM for δ-MnO_2_-only, δ-MnO_2_ + lepidocrocite, and δ-MnO_2_ + 2-line ferrihydrite, respectively. Like in the manganite systems, the rates and the concentrations of Mn-DFOB mobilized at pH 7.0 and pH 7.5 were similar (Figure S5b and Table S4).

#### 3.1.2 Fe-DFOB Mobilization from Fe(III) Oxyhydroxides: Effect of Mn(III, IV) Oxyhydroxides

In addition to Mn mobilization by DFOB, Fe mobilization from lepidocrocite and 2-line ferrihydrite was monitored in the kinetic experiments in order to assess the potential interference of Mn (both Mn(III,IV) oxyhydroxide and Mn-DFOB) on Fe mobilization by DFOB (Figure 1c,d). In the lepidocrocite-only treatment, the Fe-DFOB concentration reached the maximum possible concentration of 50 μM after 72 h (Figure 1c). For the first 2 h, the initial Fe-DFOB mobilization rate (0.018 mol kg^–1^ h^–1^) in the lepidocrocite + manganite treatment was comparable to the initial Fe-DFOB mobilization rate (0.022 mol kg^–1^ h^–1^) in the lepidocrocite-only treatment, indicating that manganite does not affect the initial kinetics of Fe-DFOB mobilization. However, after 2 h, the Fe-DFOB mobilization rate in the mixed mineral treatment slowed down. Additionally, the Fe-DFOB concentration measured at 72 h (22.2 μM) was a factor of 2.3 smaller than in the lepidocrocite-only treatment. In the treatment with added δ-MnO_2_, the initial mobilization rate of Fe-DFOB (0.0046 mol kg^–1^ h^–1^) and the maximum concentration of Fe-DFOB (1.6 μM) were lower by a factor of 4.8 and 31.2, respectively, than in the lepidocrocite-only system (Table S4).

In comparison to the lepidocrocite treatments, the Mn(III,IV) oxyhydroxides had a smaller effect on Fe-DFOB mobilization from 2-line ferrihydrite due to the fast kinetics of Fe-DFOB mobilization relative to Mn-DFOB mobilization. The initial Fe-DFOB mobilization rates from 2-line ferrihydrite were suppressed by a factor of 1.2 and 3.1 in the presence of manganite and δ-MnO_2_, respectively (Figure 1d and Table S4). In addition, the highest Fe-DFOB concentration in the 2-line ferrihydrite-only treatment was 38 μM, which was smaller than the added DFOB concentration. This is due to the adsorption of DFOB (estimated adsorbed DFOB: 7 μM) (Figure S9d) and (re)adsorption of Fe-DFOB (estimated adsorbed Fe-DFOB: 4.3 μM) (Figure S11d). In the mixed mineral systems, the Fe-DFOB concentrations reached maximum values 31.2 and 12.4 μM in the manganite +2-line ferrihydrite and δ-MnO_2_ + 2-line ferrihydrite systems, respectively. The mobilized Fe-DFOB concentrations in the mixed mineral treatments were low but remained constant for 72 h, suggesting that the Fe-DFOB complex has higher stability than Mn-DFOB even in the presence of δ-MnO_2_.

#### 3.1.3 Metal-DFOB Mobilization in Mixed Mineral Systems

Figure 1e shows the increase in metal-DFOB complexes over time for the treatments containing manganite. The kinetics and extent of metal-DFOB mobilization were similar in the manganite-only and lepidocrocite + manganite treatments but slower for the 2-line ferrihydrite + manganite treatment. Additionally, the metal-DFOB concentration measured at 72 h in these treatments accounted for only up to 76% of the added DFOB, suggesting a loss of more than 24% in the chelation ability of the added DFOB. In the treatment systems containing δ-MnO_2_, the total metal-DFOB concentrations accounted for only up to 25% of the added DFOB, a factor of 3 lower than in the manganite systems (Figure 1f). Figure 1f also shows that the metal-DFOB concentrations were greater in the mixed mineral treatments than in the δ-MnO_2_-only treatment, such that the metal-DFOB concentration increased from 1.0 to 1.6 and 12.6 μM for δ-MnO_2_-only, lepidocrocite + δ-MnO_2_, and 2-line ferrihydrite + δ-MnO_2_. Instead, the metal-DFOB concentrations decreased over time in the treatments that did not contain ferrihydrite (i.e., δ-MnO_2_-only, lepidocrocite + δ-MnO_2_) due to the oxidative decomposition of metal–ligand complexes, as discussed further in Section 4.2.

### 3.2 Reductive Dissolution of Mn(III,IV) Oxyhydroxides: Effect of Fe(III) Oxyhydroxide Minerals

**Figure 2:**
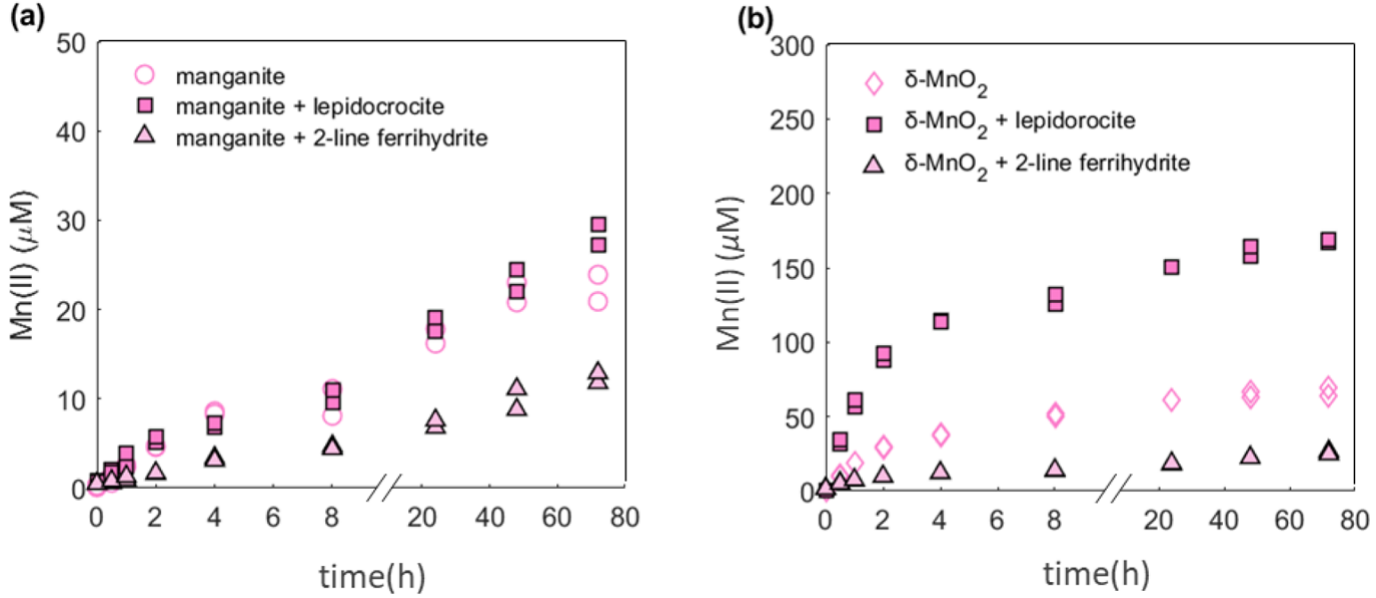
Manganese(II) mobilization by 50 μM DFOB from (a) manganite and (b) δ-MnO_2_ as a function of time at pH 7.0 under oxic conditions in the presence and absence of lepidocrocite and 2-line ferrihydrite (1 mM Mn, 1 mM Fe, 0.1 M NaCl). Note the difference in the *y*-axis scale for manganite (a) and δ-MnO_2_ (b).

In all treatments containing Mn(III,IV) oxyhydroxides, the maximum metal-DFOB concentrations were lower than the added DFOB concentration (Figure 1e,f) and the Mn(II) concentration increased over time (Figure 2), indicating that a fraction of the added DFOB or metal-DFOB was oxidized by the Mn(III,IV) oxyhydroxides. In contrast, control experiments where manganite and δ-MnO_2_ were reacted without DFOB at pH 7.0 showed negligible proton-promoted reductive dissolution (Figure S4a) with less than 2.0 and 0.5 μM Mn(II) measured in solution after 72 h of reaction.

In the manganite-only treatment, the dissolved Mn(II) concentration increased over time after DFOB addition, albeit at an initial rate that was 7 times smaller than the Mn-DFOB mobilization rate (Figure 2a and Table S4). Further, unlike the Mn-DFOB concentration, which plateaued after 4 h, the Mn(II) concentration increased slowly but continuously to a concentration of 22.4 μM over the course of 72 h. In the presence of lepidocrocite, the Mn(II) mobilization rate (0.028 mol kg^–1^ h^–1^) and total mobilized Mn(II) concentration were higher than in the manganite-only treatment. In the presence of 2-line ferrihydrite, instead, the Mn(II) mobilization rate (0.0058 mol kg^–1^ h^–1^) was 3.8 and 4.8 times lower than in the manganite-only and manganite + lepidocrocite systems, respectively. The lower Mn(II) mobilization rate in the 2-line ferrihydrite + manganite treatment can be explained by the enhanced adsorption of DFOB onto the 2-line ferrihydrite (Figure S9d) and faster kinetics of Fe-DFOB mobilization compared to those for Mn-DFOB (Figure S7b), which makes DFOB unavailable for complexation by manganite. At pH 7.5, the Mn(II) mobilization rates were slower than at pH 7.0 due to readsorption and subsequent re-oxidation of Mn(II) (Figure S6).

In treatments containing δ-MnO_2_, the addition of DFOB generated Mn(II) as the dominant dissolved metal species (Figure 2b). In the δ-MnO_2_-only treatment, the initial mobilization rate of Mn(II) (0.14 mol kg^–1^ h^–1^) was 5 times higher than the initial mobilization rate for Mn-DFOB. In the presence of lepidocrocite, both the Mn(II) mobilization rate (0.42 mol kg^–1^ h^–1^) and the maximum Mn(II) concentration (167 μM) were 3 times larger than in the δ-MnO_2_-only treatment, suggesting that the DFOB reacted with δ-MnO_2_ can be oxidized by lepidocrocite. In contrast, in the presence of 2-line ferrihydrite, the initial Mn(II) mobilization rate (0.038 mol kg^–1^ h^–1^) was 4 times smaller than in the δ-MnO_2_-only treatment (Table S4), as expected based on the faster kinetics of DFOB complexation of Fe than Mn. In comparison to the treatments at pH 7.0, the mobilization rates of Mn(II) were consistently lower at pH 7.5.

### 3.3 Fate of DFOB

**Figure 3:**
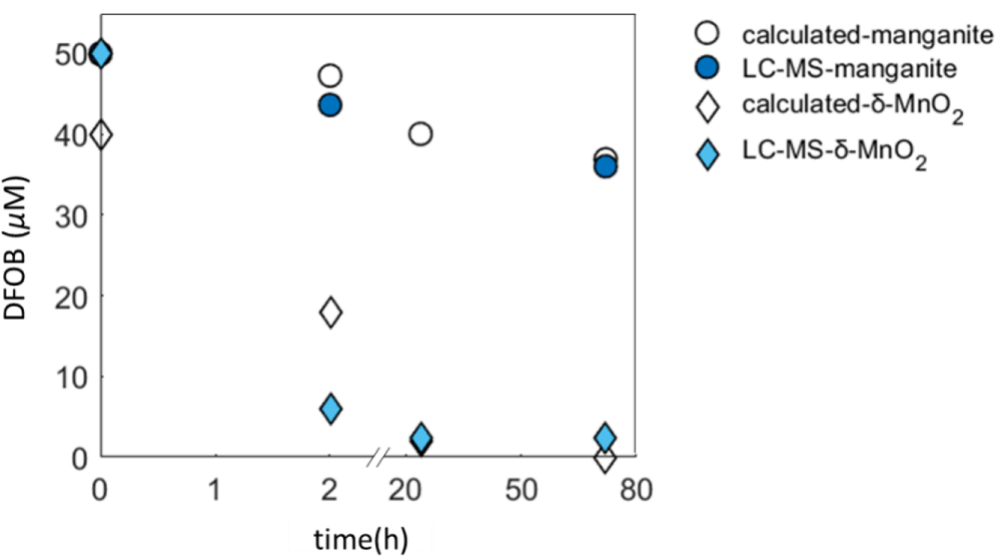
Changes in DFOB concentration (DFOB_[tot]_ = [DFOB] + [Mn-DFOB]) after reaction with manganite and δ-MnO_2_ (1 mM Mn, 0.1 M NaCl) as a function of time at pH 7.0 under oxic conditions as calculated by mass balance or measured directly by LC–MS measurements. In both treatments, the DFOB concentration at *t* = 0 was 50 μM.

Changes in the DFOB concentration were measured for a subset of samples in the manganite-only and δ-MnO_2_-only treatments as presented in Figure 3. The aqueous concentrations of total DFOB (DFOB_[tot]_ = [DFOB] + [Mn-DFOB]) calculated by mass balance are generally consistent with the LC–MS analysis for a subset of samples. For the manganite-only treatment, LC–MS analysis showed 36 μM Mn-DFOB, which indicates that of the added DFOB, about 70% acted as a Mn(III) binding ligand and 30% acted as a reducing agent. Given that 14 μM DFOB was oxidized and 26 μM Mn(II) was generated (22 μM dissolved Mn(II) (Figure 2a) + 3.6 μM adsorbed Mn(II) (Figure S8a)), we estimated that on average 2 moles of electrons were transferred to manganite per mole of DFOB (Figure 3). In the δ-MnO_2_-only treatment, at most 10% of the added DFOB formed Mn-DFOB, albeit for a short period of time (Figure 1b). Based on the maximum mobilized Mn(II) concentration in the δ-MnO_2_-only treatment (69 μM Mn(II), Figure 2b, 2 moles of electrons transferred from DFOB to Mn(IV) per mole of Mn(II) generated) and solid-phase Mn(III) concentration (50 μM, Figure 3, 1 mole of electron transferred from DFOB to Mn(IV)), we estimated that DFOB and/or DFOB oxidation products transferred approximately four electrons to Mn(IV) or Mn(III) in δ-MnO_2_. These Mn(II) an Mn(III) concentrations were corrected based on the measurement of reduced Mn in separate experiments using δ-MnO_2_ and the mesylate salt present with the DFOB compound. We found that mesylate can generate up to 15 μM solid-phase Mn(III) but no Mn(II). Finally, the discrepancy (up to 25%) between calculated and measured DFOB concentrations for early time points (Figure 3) may be due to the difficulty in quantifying adsorbed species, which themselves may undergo additional redox transformations.

The reaction of DFOB and manganite generated oxidized DFOB that gained an oxygen atom (577.3578 *m*/*z*, (561.3595 *m*/*z* (DFOB) + 15.9983 *m*/*z* (O)) as well as smaller molecules (around 300 *m*/*z*), however the intensities of these molecules were small (Figure S13). Instead, the reaction of DFOB and δ-MnO_2_ generated various oxidation products, including smaller molecules (less than 100 *m*/*z*, Figure S14b,c) and dimers (between 715 and 831 *m*/*z*, Figure S14e,f). Neither acetate nor succinate was detected in our LC–MS analyses, in contrast to previous studies that have proposed that DFOB can be oxidized to succinate and acetate by pyrolusite^64^ and to acetate by goethite.^65^

### 3.4 Fate of Mobilized Mn-DFOB and Mn(II) in Mixed Mineral Systems

**Figure 4:**
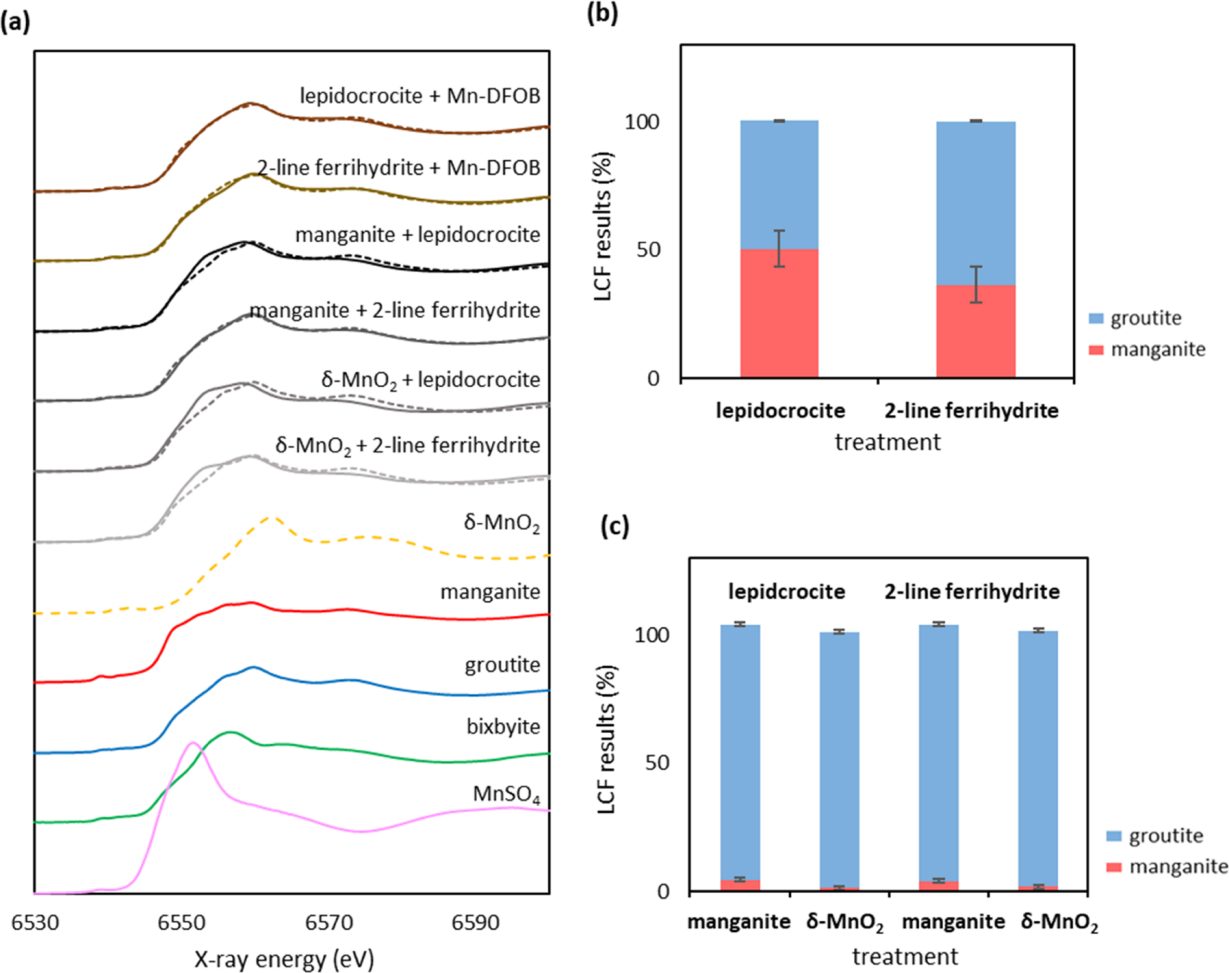
Manganese K-edge XANES spectra of (a) reference samples (MnSO_4_, bixbyite, groutite, manganite, and δ-MnO_2_) and experimental samples (1 mM Mn-DFOB added to 10 mM lepidocrocite and 10 mM 2-line ferrihydrite, and 1 mM DFOB added to 10 mM of lepidocrocite and 2-line ferrihydrite in the presence of manganite and δ-MnO_2_). Linear combination fit results are shown by the dashed lines. Linear combination fit results to determine manganese speciation are shown in (b) for a sample where Mn-DFOB was added to Fe(III) oxyhydroxides and (c) for samples where DFOB was added to mixed mineral systems. The fitting error for each component is plotted as an error bar.

To examine the fate of Mn-DFOB and Mn(II) formed from the interaction of manganite and δ-MnO_2_ with DFOB, we measured the Mn K-edge XA spectra from a subset of samples. The Mn K-edge XANES and EXAFS spectra from the experimental samples were compared to the spectra measured from δ-MnO_2_, manganite, groutite (α-MnOOH), bixbyite (Mn_2_O_3_), and aqueous Mn(II) (MnSO_4_) references (Figure 4a). The Mn K-edge XANES spectra for the experimental samples and linear combination fits (LCFs) based on reference spectra are shown in Figure 4a and summarized in Figure 4b,c. The LCFs reproduced the experimental spectra with component sums of 100–104% and *R* factors <6.6 × 10^–3^, showing that all Mn associated with lepidocrocite and ferrihydrite was Mn(III) (Table S7). Thus, the interaction of Mn-DFOB with Fe(III) oxyhydroxides and interaction of DFOB with Mn(III,IV) oxyhydroxides and Fe(III) oxyhydroxides can redistribute Mn from Mn(III,IV) oxyhydroxides to Fe(III) oxyhydroxides.

In Type 1 samples, where we examined the fate of Mn-DFOB after the Mn-for-Fe metal exchange reaction, we found that the added 1 mM Mn-DFOB decomposed fully after 24 h (Figure S15) and that Fe-DFOB increased to 296 μM for lepidocrocite and 644 μM for 2-line ferrihydrite and then remained constant over 10 days. These results indicate that 30% (lepidocrocite) and 64% (2-line ferrihydrite) of the added Mn-DFOB underwent a Mn-for-Fe exchange reaction and that the remaining Mn-DFOB underwent oxidative decomposition. Of the 1 mM Mn-DFOB added, 261 and 188 μM Mn were measured as dissolved Mn(II) in the lepidocrocite and 2-line ferrihydrite treatments, respectively. The remaining Mn precipitated onto the Fe(III) oxyhydroxide minerals as Mn(III). The Mn K-edge XANES spectra of these samples were most similar to manganite and groutite (Figure 4b and Table S7).

In Type 2 samples, where DFOB was added to the mixed-mineral treatments using the Float-a-lyzer devices, we found that the added 1 mM DFOB increased Mn-DFOB concentration up to 650 μM from manganite and up to 115 μM from δ-MnO_2_. However, the mobilized Mn-DFOB concentrations decreased over time after they reached the maximum concentrations (Figure S16a,b), which suggests that the Mn-DFOB complexes decomposed, forming Mn(II) (Figure S16e,f) and/or Mn precipitates. The spectra collected from the Mn precipitates associated with the Fe(III) oxyhydroxides were most similar to the groutite reference spectrum irrespective of the treatment type (Figure 4c), which is consistent with previous research.^66^ Thus, we found no relationship between short-range order of the Mn surface precipitates (<6 Å) and the host Fe oxyhydroxide phase. In other words, the Mn(III) surface precipitates were not isostructural with the host Fe phase (i.e., manganite onto lepidocrocite or hematite onto 2-line ferrihydrite). Consistent with the XANES analysis, the Mn K-edge EXAFS spectra matched most closely to the EXAFS spectrum from groutite (Figure S17a) although the LCFs were of lower quality with component sums of 82–95% and *R*-factor values of less than 2.5 × 10^–1^ (Table S8). Inspection of the Fourier transformed Mn K-edge EXAFS spectra shows that the mismatch between the experimental samples and reference minerals occur mainly at *R* + Δ*R* > 4 Å (Figure S17b). Together, these results suggest that the Mn(III) precipitates associated with the Fe(III) oxyhydroxides are less ordered than the reference minerals.

## 4 Discussion

### 4.1 Synthesis of Mn and Fe Dissolution Rates

**Figure 5:**
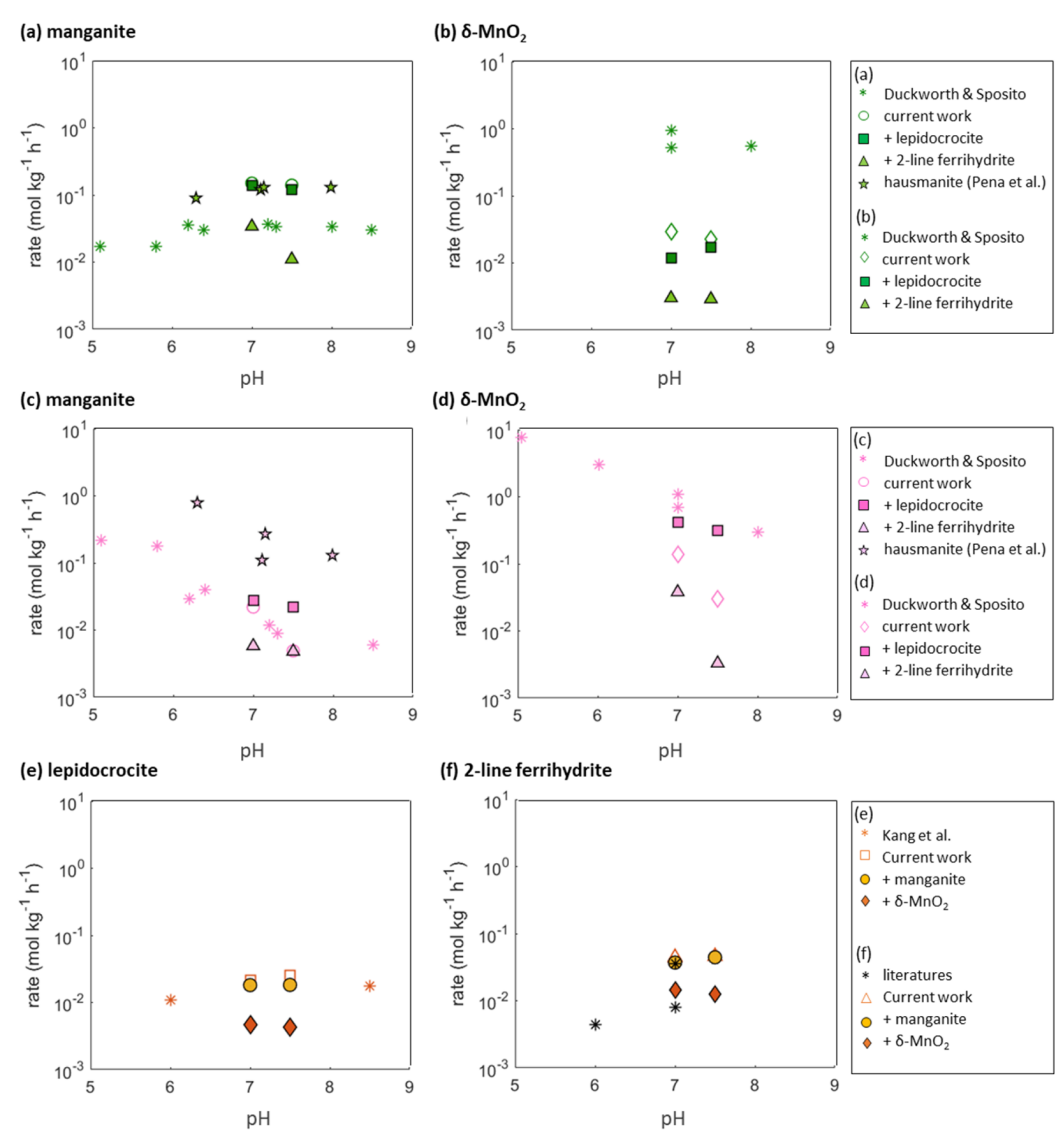
Synthesis of measured initial mobilization rates for (a and b) Mn-DFOB, (c and d) Mn(II), and (e and f) Fe-DFOB from (a and c) manganite, (b and d) δ-MnO_2_, (e) lepidocrocite, and (f) 2-line ferrihydrite from current work and literature values. Current work: 1 mM Mn(III,IV) or Fe(III) oxyhydroxides, 0.1 M NaCl, and 50 μM DFOB. Duckworth and Sposito: 0.1 g L^–1^ δ-MnO_2_ and 0.7 g L^–1^ manganite, 0.1 M NaCl, 10–20 mM acetate, MES, MOPS, and HEPES, and 100 μM DFOB. Peña et al.: 2 g L^–1^ hausmannite, 0.1 M NaCl, 30 mM acetate, MES, MOPS, and HEPES buffer, and 100 M DFOB. Kang et al.: 0.1 g L^–1^ lepidocrocite and 2-line ferrihydrite, 0.01 M NaCl, 5 mM MES, MOPS, and PEPES buffers, and 20 μM DFOB. Mikutta and Kretzschmar: 0.5 g L^–1^ 2-line ferrihydrite, 0.01 M NaClO_4_, and 10 μM DFOB. Poggenburg et al: 0.2 g L^–1^ 2-line ferrihydrite, 0.01 M KCl, and 100 μM DFOB.

In Figure 5, we compare the measured rates of Mn-DFOB, Mn(II), and Fe-DFOB mobilization to published values. The Mn-DFOB mobilization rates from manganite were consistent with published values for hausmannite^45^ and manganite^22^ (Figure 5a), but an order of magnitude smaller than reported previously for δ-MnO_2_ and biogenic MnO_2_ by Duckworth and Sposito^76^ (Figure 5b). The major difference between the work of Duckworth and Sposito and our work is their use of organic buffers to control pH (20 mM HEPES and MOPS). Organic buffers, such as HEPES, can reduce Mn(IV) in δ-MnO_2_ to Mn(III) and lead to the formation of Mn(III)-rich δ-MnO_2_.^60,77^ The mobilization of Mn-DFOB from Mn(III)-rich δ-MnO_2_ (≤3.65)^78^ would be more favorable than mobilization of Mn-DFOB from δ-MnO_2_ (AMON = 4) because *a priori* reduction of Mn(IV) is not required and because the lower reduction potential of Mn(III)-rich δ-MnO_2_^79,80^ limits the oxidative decomposition of DFOB and Mn-DFOB.

While the Mn-DFOB mobilization rates did not vary with pH, the Mn(II) mobilization rates decreased with increasing pH (Figure 5c,d). The slower mobilization of Mn(II) with increasing pH can be attributed to the slower decomposition of Mn-DFOB,^14^ slower rate of manganite reduction,^22^ and enhanced adsorption and possible reoxidation of Mn(II) onto both Mn and Fe mineral surfaces (Figure S8). These rates are consistent with those published for manganite (Duckworth and Sposito, 2005)^22^ but are an order of magnitude smaller than those for hausmannite due to the presence of Mn(II) in hausmannite (AMON of 2.67) (Peña et al., 2007)^45^ and the higher solubility of hausmannite compared to manganite.^81^ We also found that Mn(II) mobilization from δ-MnO_2_ was 5 times smaller than previously reported.^76^ We expect that the higher rates reported previously can be explained by presence of Mn(III) in δ-MnO_2_ upon reaction with HEPES.^79^ This suggests that the reduction of Mn(III) rather than the detachment of surface-associated Mn(II) is the rate limiting step in Mn(II) mobilization.

For the single Fe(III) oxyhydroxide mineral systems, the measured Fe-DFOB mobilization rates were in agreement with published values for lepidocrocite (Figure 5e)^47^ and varied slightly for 2-line ferrihydrite (Figure 5f).^47,82,83^ This small variation may arise from differences in the experimental design, including pH control, electrolyte concentration, and presence of oxygen. In the presence of Mn oxyhydroxides, we observed lower rates of DFOB-promoted dissolution of Fe oxyhydroxides, especially over short reaction times (<8 h). This result suggests that the Fe mobilization rates may be lower in natural environments where Fe and Mn oxyhydroxide minerals co-occur.

### 4.2 Mn and Fe Mobilization Mechanism by DFOB in Mixed Mineral Systems

**Figure 6:**
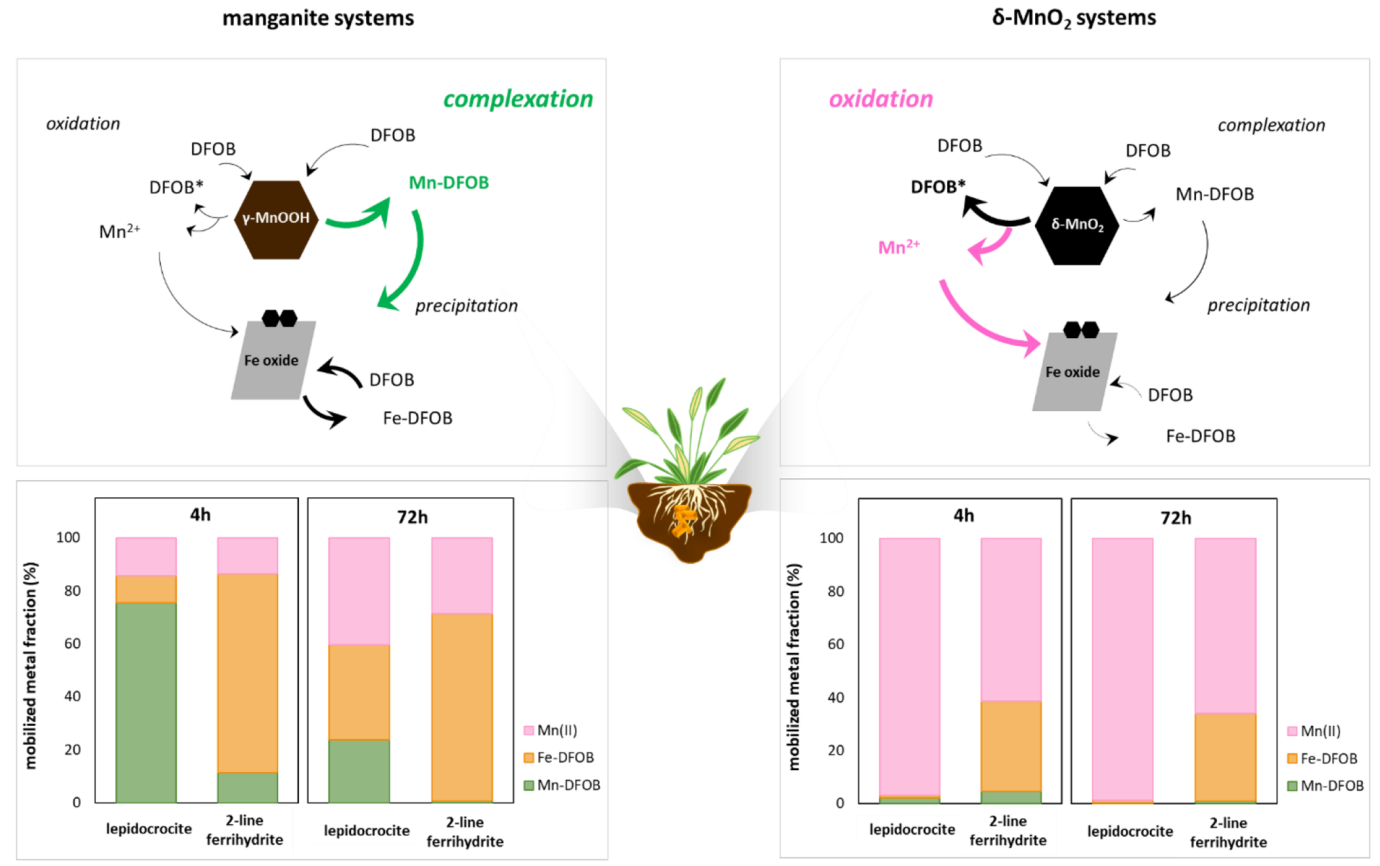
Schematic figure that describes the results from current experimental systems. The fraction (%) of each species mobilized by DFOB was calculated for 4 and 72 h.

A schematic showing the mechanisms of Fe and Mn mobilization in mixed mineral systems is presented in Figure 6. As discussed below, the dominant metal mobilization mechanism varied based on the combination of mineral, such that ligand-promoted dissolution was dominant in the manganite systems and reductive dissolution was dominant in the δ-MnO_2_ systems.

#### 4.2.1 Manganite Systems

In the treatments containing manganite, ligand-promoted dissolution was the dominant metal mobilization mechanism (Figure 6). In the manganite-only treatment, at most 76% (38 μM) of the added DFOB formed Mn-DFOB complexes (Figure 1a) despite the fact that DFOB can bind Mn(III) in a 1:1 ratio.^44^ This result is consistent with previous studies, which showed that about 75% of the added DFOB formed Mn-DFOB from manganite^22^ and hausmannite.^45^ The amount of Mn-DFOB mobilized from manganite at short times, however, was much lower with added 2-line ferrihydrite than added lepidocrocite.

Lepidocrocite and manganite are isostructural and have similar specific surface areas (Table S2). However, the solubility of lepidocrocite (log *K*_sp_: 1.37) is substantially lower than the solubility of manganite (log *K*_sp_: 4.57), indicating greater lability of Mn than Fe upon DFOB adsorption. Additionally, DFOB can be oxidized by the manganite surface, which generates surface Mn(II).^22^ Any Mn(II) on the manganite surface potentially increases metal lability through electron delocalization between structural Mn(III) and Mn(II).^84^ Greater lability of Mn(III) species associated with higher solubility of manganite as well as catalytic effect of electron excess on the mineral surface may govern the initial kinetics of ligand-promoted dissolution in the lepidocrocite and manganite mixed mineral system. On the other hand, the solubility of 2-line ferrihydrite (log *K*_sp_: 4.89) and manganite are similar, but 2-line ferrihydrite has a higher specific surface area than manganite (Table S2). The larger specific surface area allows for 3 times more DFOB adsorption by 2-line ferrihydrite than manganite (Figure S9), thereby limiting the access of DFOB to the manganite surface and hindering the ligand-promoted dissolution of manganite. In both mixed-mineral systems, the mobilized Mn-DFOB concentration decreased over time due to the metal exchange reaction with Fe(III) oxyhydroxides (Figure 6).

Unlike Mn-DFOB, the Fe-DFOB concentration either increased continuously during 72 h (lepidocrocite) or remained constant once the maximum concentration was attained (2-line ferrihydrite) (Figure 1c). Thus, Fe-DFOB complexes are stable against metal exchange reactions and/or oxidative decomposition by manganite. The lack of oxidative decomposition of Fe-DFOB can be explained by the lower reduction potential of the Fe-DFOB couple compared to Mn-DFOB, which renders Fe-DFOB stable in the presence of manganite (Figure S1).

In the manganite systems, the Mn(II) concentration increased slowly but continuously over the course of the experiment (Figure 2a). A similar trend was observed by Duckworth and Sposito (2005).^22^ These results suggest that oxidative decomposition of Mn-DFOB is more likely than oxidation of DFOB or oxidized DFOB fragments. Additionally, the total mobilized Mn(II) increased further in the presence of lepidocrocite, which may result from the lepidocrocite-induced decomposition of Mn-DFOB. Specifically, upon the adsorption of Mn-DFOB to lepidocrocite (Figure S10c), DFOB can participate in a metal exchange reaction given the higher stability constant of Fe-DFOB (log *K*: 33.0^47^) than Mn-DFOB (log *K*: 29.0^14^). Any Mn(III) liberated from the metal-exchange reaction can generate Mn(II) either through disproportionation of Mn(III) or reduction of Mn(III) to Mn(II) coupled to ligand oxidation.

#### 4.2.2 δ-MnO_2_ Systems

In the treatments containing δ-MnO_2_, one of the strongest oxidants in aquatic and terrestrial systems,^85,86^ the dominant metal mobilization mechanism involved reductive dissolution of δ-MnO_2_ (Figure 6) coupled to the oxidation of DFOB and Mn-DFOB. Due to the initial loss of DFOB by δ-MnO_2_, at most, 25% of the added DFOB was able to form metal-DFOB complexes in the δ-MnO_2_ systems. Additionally, the Mn-DFOB concentrations decreased substantially from their maximum values, even in δ-MnO_2_-only system, confirming that Mn-DFOB is unstable in the presence δ-MnO_2_. Through LC–MS analyses, we found (i) that δ-MnO_2_ destroys the hydroxylamine functional groups, which results in small organic fragments with no metal-binding capacity (Figure S14b,c) and (ii) evidence for dimerization of the DFOB oxidation products (Figure S14d,e). In all δ-MnO_2_ containing treatments, the remaining Mn-DFOB concentrations were in the submicromolar range, which are consistent with the concentration of aqueous Mn(III) detected in natural environments.^30,33,34,36,37^

In the δ-MnO_2_ systems, the initial Fe-DFOB mobilization rates and the total mobilized Fe-DFOB concentration decreased substantially due to the oxidative loss of DFOB but remained constant once formed, as expected since Fe-DFOB is stable against oxidative decomposition by Mn(III,IV) oxyhydroxides. The stability of Fe-DFOB is likely due to the marginal adsorption of Fe-DFOB onto the Mn(III,IV) oxyhydroxides (Figure S11a,b) and lower redox potential of the Fe(III)-DFOB/Fe(II)-DFOB (*E*_1/2_ in V vs NHE: −0.448)^87^ couple relative to the manganite/Mn^2+^ and δ-MnO_2_/Mn^2+^ couple (Figure S1).

For Mn(II), we observed higher rates than for Mn-DFOB and/or Fe-DFOB mobilization (Figures 1 and 2). This result suggests that Mn(II) formation results more likely from the direct reduction of δ-MnO_2_ by DFOB rather than Mn-DFOB. Because the δ-MnO_2_ used in our study had an initial AMON value of 4.01 (Table S2), surface Mn(III) is likely an intermediate that forms prior to the mobilization of Mn(II).^78^ Both AMON titrations and PP-extractions showed 10% Mn(III) in δ-MnO_2_ after interaction with DFOB (Table S5). Subsequent reduction of Mn(III) by DFOB, disproportionation of Mn(III) to Mn(II) and Mn(IV) and/or reduction of Mn(III) coupled to further oxidation of DFOB fragments can all lead to production of aqueous Mn(II). The presence of lepidocrocite also accelerated the decomposition of Mn-DFOB and thus the generation of dissolved Mn(II), while the 2-line ferrihydrite + δ-MnO_2_ treatment showed the lowest Mn(II) concentration due to the facile formation of Fe-DFOB (Figure S7d).

## 5 Conclusions

In this study, we examined metal–ligand complex formation, extent of ligand competition, and decomposition of ligand and metal–ligand complexes in mixed mineral systems containing Mn(III,IV) and Fe(III) oxyhydroxides. We showed that siderophores can increase aqueous Mn(III) concentrations in mixed-mineral systems, although the mechanism of Mn-DFOB formation varies with the type of Mn(III,IV) oxyhydroxide minerals. For manganite, Mn-DFOB complexes formed by ligand-promoted dissolution; while for δ-MnO_2_, Mn-DFOB complexes formed upon reduction of surface Mn(IV) to Mn(III) by DFOB and subsequent detachment of Mn(III) by unoxidized DFOB. Importantly, we observed that the initial kinetics of metal mobilization were governed by Mn mobilization (Mn-DFOB and Mn(II)) rather than Fe mobilization. However, the mobilized Mn-DFOB decreased over time due to metal exchange reactions or oxidation reactions by Mn and Fe mineral surfaces. Our results demonstrate that siderophores can lead to outbursts of short-lived Mn(III) species, affecting the function of siderophores as well as Fe acquisition efficiency. This is of particular relevance to natural systems like the rhizosphere where soil minerals and root exudates co-exist.

Iron acquisition strategies that rely on siderophore exudation can be significantly hindered by the presence of Mn(III,IV) oxyhydroxides. Our observations of Mn-DFOB concentrations in the δ-MnO_2_ + 2-line ferrihydrite treatment are consistent with the nano- to submicromolar aqueous Mn(III) concentrations concentrations commonly observed in terrestrial and aquatic environments,^37^ where birnessite-like minerals and ferrihydrite are prevalent. Although the Mn-DFOB concentrations were low, we observed that Mn(III,IV) oxyhydroxides can limit Fe-DFOB mobilization due not only to competition with Mn(III) but also surface-catalyzed oxidative decomposition of DFOB. Siderophore decomposition is particularly significant in the context of biological Fe acquisition strategies. When plants and microbes experience Fe deficiency, they exude both siderophores and reductants. Reductant exudation can lower the redox potential of Mn(III,IV) oxyhydroxides and increase the solubility of both Mn(III) and Fe(III) without compromising the integrity of the siderophores. Further research is needed to evaluate this mechanism. Finally, because siderophores can increase both Mn(III) and Fe(III) solubility, the classification of siderophores solely as Fe binding ligands should be re-evaluated.

Our Mn K-edge XANES and EXAFS spectra showed the formation of Mn(III)-bearing precipitates on Fe(III) oxyhydroxides surfaces. The redistribution of Mn from Mn(III,IV) oxyhydroxides to Fe(III) oxyhydroxides, which was mediated through ligand-promoted and reductive dissolution of the Mn mineral and subsequent readsorption of Mn-DFOB and Mn(II), shows that siderophores can act as manganese vectors. This finding is relevant in various environments. For instance, siderophores have been implicated in the formation of Fe–Mn nodules in sediments in marine environments.^88−90^ Furthermore, although Mn abundance is an order of magnitude lower than Fe abundance in soil, the impacts of Mn chemistry in soil biogeochemical cycles can be similar or greater than that of Fe. Our data suggest that siderophore-mediated redistribution of Mn can shift the surrounding redox environment toward more oxidizing conditions. In particular, Mn-coated/incorporated Fe(III) oxyhydroxides may have distinct sorption and oxidation properties from pure Fe(III) oxyhydroxides and thus affect the mobility and speciation of trace metal nutrients and contaminants as well as organic carbon.^91^

## Supporting Information Available

Tables of chemicals used for this study, properties of synthetic Mn and Fe oxyhydroxides, extinction coefficient for Fe-DFOB and Mn-DFOB interferences, summary of experiments and mobilization rates, summary of AMON values and PP extracted Mn(III) concentrations, summary of adsorption experiments and the *q*_max_ and *K*_D_ values, and linear combination fitting for Mn K-edge XANES and EXAFS spectra, figures of redox ladder for Fe, Mn, and DFOB species, XRD patterns, absorbance of metal–ligand complexes under various conditions, adsorption isotherms, summary of the batch dissolution experiments, dissolution rates and decomposition coefficients, AMON and PP-extraction data, dissolution data at pH 7.5, LC–MS data, and wet chemistry data for XAS samples and EXAFS data, and discussion of procedure for calculating Fe-DFOB and Mn-DFOB interference (PDF)
