# Supplementary material for: Siderophore-Mediated Mobilization of Manganese Limits Iron Solubility in Mixed Mineral Systems
Source: ACS Earth Space Chem. 2023 Mar 9;7(4):662–75. doi: 10.1021/acsearthspacechem.2c00271 (PMC10123812; doi:10.1021/acsearthspacechem.2c00271)
Supplement: Supplementary file 1 — sp2c00271_si_001.pdf [file sp2c00271_si_001.pdf]

Supporting Information

Siderophore-mediated mobilization of manganese limits iron solubility in mixed mineral systems

Kyounglim Kang[1*] and Jasquelin Peña[1,2*]

[1]Department of Civil and Environmental Engineering, University of California, Davis, California 95616, United States

[2]Energy Geosciences Division, Lawrence Berkeley National Laboratory, Berkeley, California 94720, United States

Corresponding Author*

klkang@ucdavis.edu and pena@ucdavis.edu

Supporting Information

Content

Tables	*contents*		*page*
**Table. S1.**	List of chemicals used for this study	....	4
**Table. S2.**	Physicochemical properties of synthetic Mn and Fe oxyhydroxides	....	5
**Table. S3.**	Extinction coefficient for Fe-DFOB and Mn-DFOB interferences	....	5
**Table. S4.**	Summary of experiments and corresponding initial metal mobilization rates	....	6
**Table. S5.**	Summary of AMON values and PP extracted Mn(III) concentrations from Mn(III, IV) oxyhydroxide minerals	....	7
**Table. S6.**	Summary of adsorption experiments and the q_{max} and K_D values	....	8
**Table. S7.**	Linear combination fitting results for Mn K-edge XANES spectra	....	9
**Table. S8.**	Linear combination fitting results for Mn K-edge EXAFS spectra	....	9
Figures			
**Figure. S1.**	Redox ladder for Fe, Mn and DFOB species	....	10
**Figure. S2.**	XRD patterns of Mn oxides and Fe oxides	....	11
**Figure. S3.**	Absorbance of metal-ligand complexes	....	12
**Figure. S4.**	Mn mobilization from manganite and δ-MnO_2 in the absence of DFOB	....	14
**Figure. S5.**	Mn and Fe mobilization from Mn(III,IV) oxyhydroxides in the presence and absence of Fe(III) oxyhydroxides by DFOB at pH 7.5	....	15
**Figure. S6.**	Mn(II) mobilization by DFOB from Mn(III,IV) oxyhydroxides in presence and absence of Fe(III) oxyhydroxides on a linear scale	....	16
**Figure. S7.**	Mn-DFOB, Fe-DFOB and Mn(II) mobilization on a logarithmic scale	....	17
**Figure. S8.**	Adsorption isotherms for Mn(II) onto various Mn(II(,IV) and Fe(III) oxyhydroxide minerals	....	18

Figure. S9. Adsorption isotherms for DFOB onto various Mn(II(,IV) and Fe(III) oxyhydroxide minerals 19

Figure. S10. Adsorption isotherms for Mn-DFOB onto various Mn(II(,IV) and Fe(III) oxyhydroxide minerals 20

Figure. S11. Adsorption isotherms for Fe-DFOB onto various Mn(II(,IV) and Fe(III) oxyhydroxide minerals 21

Figure. S12. LC-MS analysis of 50 µM DFOB 22

Figure. S13. LC-MS analysis of DFOB degradation products after reaction with manganite 23

Figure. S14. LC-MS analysis of DFOB degradation products after reaction with δ-MnO_2 24

Figure. S15. Wet chemical data for sample type 1 27

Figure. S16. Wet chemical data for sample type 2 28

Figure. S17. Mn K-edge EXAFS and fourier transform of the Mn K-edge EXAFS spectra 29

Text

Text. S1. Mn-DFOB and Fe-DFOB concentration calculation 30

References 31

Tables

Table S1. List of chemicals used for this study.

Chemical	Formula	Supplier	CAS number	Purity
Manganese(II) sulfate monohydrate	$MnSO_4 \cdot H_2O$	Millipore sigma	10034-96-5	≥99.0%
Hydrogen peroxide solution	H_2O_2	Millipore sigma	7722-84-1	30.0% (W/W) in H_2O
Ammonia solution 25%	NH_4OH	Millipore sigma	7336-21-6	30.0% (W/W) in H_2O
Iron(II) chloride tetrahydrate	$FeCl_2 \cdot 4H_2O$	Millipore sigma	13478-10-9	≥99.0%
sodium hydroxide solution	NaOH	Supelco	1310-73-2	1 M in H_2O
Hydrochloric acid solution	HCl	Supelco	7647-01-0	1 M in H_2O
Sodium chloride	NaCl	Millipore sigma	7647-14-5	≥99.0%
Iron(III) nitrate nonahydrate	$Fe(NO_3)_3 \cdot 9H_2O$	Millipore sigma	7782-61-8	≥99.95%
Manganese(II) chloride	$MnCl_2$	Millipore sigma	7773-01-05	≥99.%
mesylate salt of desferrioxamine B	$C_{25}H_{48}N_6O_8 \cdot CH_4O_3S$	Novartis	138-14-7	>97%
Sodium pyrophosphate decahydrate	$Na_4P_2O_7 \cdot 10H_2O$	Millipore sigma	13472-36-1	≥99%
Manganese(III) acetate dihydrate	$(CH_3COO)_3Mn \cdot 2H_2O$	Millipore sigma	19513-05-4	≥97%
Potassium permanganate solution	$KMnO_4$	XAXOL Chemical corporation	7722-64-7	0.01 N solution
Ammonium iron(II) sulfate hexahydrate	$(NH_4)Fe(SO_4)_2 \cdot 6H_2O$	Millipore sigma	7783-85-9	99.0%
Sulfuric acid	H_2SO_4	Millipore sigma	7664-93-9	99.999%

Table S2. Properties of synthetic Mn and Fe oxyhydroxides. For N_2 BET analysis of specific surface area analysis, the mineral sample were dried under ambient air conditions prior to the measurement. For AMON titration, which were performed in triplicate, δ-MnO_2 samples were introduced as hydrated suspensions and manganite samples were introduced as dried powders.

**mineral**	**SSA (m^2g^{-1})**	**pzc**	**AMON**
*δ-MnO_2	244	2.5[1, 2]	4.01 (± 0.01)
manganite	60	8.1-8.5[3]	3.02 (± 0.02)
lepidocrocite	108	6.6[4]	
2-line ferrihydrite	385	7.9[4]	

***** Chemical formula of δ-MnO_2: $Na_{0.19}MnO_x·0.98H_2O$

Table S3. Extinction coefficient (M^{-1} cm^{-1}) for Fe-DFOB and Mn-DFOB interferences.

pH	Fe- interference $ε_{310}$
pH 7.0	521
pH 7.5	536
	Mn- interference $ε_{430}$
pH 7.0	288
pH 7.5	279

Table S4. Summary of experiments and mobilization rates of Mn-DFOB, Fe-DFOB and Mn(II) and decomposition coefficients for Mn-DFOB obtained in this study and by previous literatures. The initial mobilization rates were calculated from the slopes of linear regression lines of the dissolved concentration over time for the data points over which the increase of dissolved Mn and Fe was linear ($R^2 > 0.90$). The decomposition rate coefficient for Mn-DFOB was calculated by fitting the Mn-DFOB concentration over time to a log-linear regression, determining the first order derivative of the fitting equation ($R^2 > 0.95$).

Mn oxyhydroxide	Fe oxyhydroxide	pH	ligand	initial mobilization (time interval of 0-2 h)			Decomposition
				Mn-DFOB (mol kg^{-1} h^{-1})	Fe-DFOB (mol kg^{-1} h^{-1})	Mn(II) (mol kg^{-1} h^{-1})	Mn-DFOB (h^{-1})
1 mM	1 mM		50 µM				
	lepidocrocite	7.0	DFOB		0.022		
		7.5			0.025		
	2-line ferrihydrite	7.0			0.044		
		7.5			0.046		
manganite		7.0	DFOB	0.15		0.022	0.0
		7.5		0.14		0.0049	0.0
0.7 g L^{-1}			100 µM				comments
manganite		7.2	DFOB	0.037		0.012	Duckworth and Sposito (2005)*
		7.3		0.034		0.0090	
2 g L^{-1}			100 µM				comments
hausmanite		7.1	DFOB	0.080		0.12	Peña et al. (2007)#
hausmanite		7.2		0.14		0.13	
manganite	lepidocrocite	7.0	DFOB	0.14	0.018	0.028	0.012
		7.5		0.12	0.018	0.022	0.0093
	2-line ferrihydrite	7.0		0.034	0.037	0.0058	0.035
		7.5		0.011	0.044	0.0048	0.059
10 mM	10 mM		1 mM				
manganite	lepidocrocite	7.0	DFOB	2.6	0.43	0.74	0.0082
	2-line ferrihydrite	7.0		0.16	0.84	0.11	0.037
1 mM	1 mM		50 µM				
δ-MnO_2		7.0	DFOB	0.029		0.14	0.023
		7.5		0.023		0.030	0.025
0.1 g L^{-1}			100 µM				comments
δ-MnO_2		7.0	DFOB	0.52		1.1	Duckworth and Sposito (2007)&
		7.0		0.95		0.70	
biogenic δ-MnO_2		7.0		2.2		0.60	
		7.0		1.8		1.0	
δ-MnO_2	lepidocrocite	7.0	DFOB	0.012	0.0046	0.42	0.075
		7.5		0.017	0.0042	0.31	0.032
	2-line ferrihydrite	7.0		0.0030	0.014	0.038	0.020
		7.5		0.0029	0.013	0.0033	0.020
10 mM	10 mM		1 mM				
δ-MnO_2	lepidocrocite	7.0	DFOB	2.1	0.026	5.8	0.031
	2-line ferrihydrite	7.0		0.21	0.26	0.65	0.0022

For all experiments conducted in this study, pH was controlled by acid or base addition on a pH STAT. For published studies, pH was controlled with the use of organic buffers. *10 mM MOPS and HEPES were used as pH buffers. #30 mM HEPES was used as a pH buffer. &20 mM MOPS and HEPES were used as pH buffers.

Table S5. Summary of AMON values and PP extracted Mn(III) concentrations from manganite and δ-MnO_2 at various experimental conditions (0.1 M NaCl). For AMON titration, δ-MnO_2 and manganite samples were introduced as a paste collected on a filter membrane.

Mn oxide	Fe oxide	pH	ligand	2 h<br>AMON<br>PP extracted Mn(III) (µM)	8 h<br>AMON<br>PP extracted Mn(III) (µM)	24 h<br>AMON<br>PP extracted Mn(III) (µM)	72 h<br>AMON<br>PP extracted Mn(III) (µM)
1 mM	1 mM		50 µM				
manganite		7.0	DFOB				3.02 (± 0.012)
manganite		7.5	DFOB				3.01 (± 0.010)
manganite	lepidocrocite	7.0	DFOB				3.02 (± 0.011)
manganite		7.5	DFOB				3.03 (± 0.017)
manganite	2-line ferrihydrite	7.0	DFOB				3.00 (± 0.013)
manganite		7.5	DFOB				3.00 (± 0.015)
δ-MnO_2		7.0	DFOB	3.95 (± 0.012)<br>40	3.90 (± 0.017)<br>69	3.89 (± 0.011)<br>70	3.89 (± 0.010)<br>75
δ-MnO_2		7.5	DFOB				3.93 (± 0.015)
δ-MnO_2	lepidocrocite	7.0	DFOB				3.92 (± 0.011)
δ-MnO_2		7.5	DFOB				3.92 (± 0.020)
δ-MnO_2	2-line ferrihydrite	7.0	DFOB				3.94 (± 0.021)
δ-MnO_2		7.5	DFOB				3.93 (± 0.027)

Table S6. Summary of adsorption experiments and the q_{max} and K_D (calculated as q/c) values calculated based on the Langmuir model.

mineral	pH	absorbent	q_{max} (μmol kg^{-1})	K_D (L μmol^{-1})
manganite	pH 7	Mn(II)	60	0.003
		DFOB	19	0.15
		Mn-DFOB	5.7	0.17
		Fe-DFOB	0.9	0.10
	pH 7.5	Mn(II)	77	0.004
		DFOB	28	0.12
δ-MnO_2	pH 7	Mn(II)	-	-
		DFOB	26	0.14
		Mn-DFOB	7.7	0.37
		Fe-DFOB	0.5	0.03
	pH 7.5	Mn(II)	-	-
		DFOB	38	0.085
lepidocrocite	pH 7	Mn(II)	99	0.001
		DFOB	132	0.01
		Mn-DFOB	65	0.05
		Fe-DFOB	0.3	0.07
	pH 7.5	Mn(II)	78	0.0027
		DFOB	115	0.02
2-line ferrihydrite	pH 7	Mn(II)	340	0.001
		DFOB	93	0.04
		Mn-DFOB	57	0.29
		Fe-DFOB	17	0.01
	pH 7.5	Mn(II)	208	0.002
		DFOB	1.9	0.03

Table S7. Linear Combination Fitting for Mn K-edge XANES spectra based on using groutite, manganite and bixbyite and $MnSO_4$ references.

Treatments	manganite (%)	groutite (%)	sum	R- factor
lepidocrocite + Mn-DFOB	50.5 (2.4)	50.1 (2.4)	1.01	9.2×10^{-4}
2-line ferrihydrite + Mn-DFOB	36.4 (2.9)	63.9 (3.0)	1.00	1.4×10^{-3}
manganite + lepidocrocite	4.6 (6.5)	99.4 (6.6)	1.04	6.6×10^{-3}
manganite + 2-line ferrihydrite	1.3 (0.2)	100 (3.7)	1.01	1.3×10^{-3}
δ-MnO_2 + lepidocrocite	4.1 (0.5)	100 (6.0)	1.04	1.5×10^{-3}
δ-MnO_2 + 2-line ferrihydrite	1.8 (0.5)	100 (2.3)	1.02	1.1×10^{-3}

Table S8. Linear Combination Fitting for Mn K-edge EXAFS spectra based on using groutite, manganite, bixbyite and $MnSO_4$ references.

Treatments	manganite (%)	groutite (%)	sum	R- factor
lepidocrocite + Mn-DFOB	18.8 (6.4)	76.3 (12.5)	0.95	0.24
2-line ferrihydrite + Mn-DFOB	35.1 (6.2)	54.6 (2.1)	0.90	0.19
manganite + lepidocrocite	1.1 (0.4)	80.9 (10.5)	0.82	0.14
manganite + 2-line ferrihydrite	3.1 (0.7)	86.8 (12.0)	0.90	0.22
δ-MnO_2 + lepidocrocite	11.4 (5.3)	82.2 (10.2)	0.94	0.18
δ-MnO_2 + 2-line ferrihydrite	5.9 (1.6)	87.5 (8.3)	0.93	0.20

Figures

Figure S1. Redox ladder for Fe, Mn and DFOB species. The redox potential values of DFOB and metal-DFOB complexes are obtained from previous literatures.

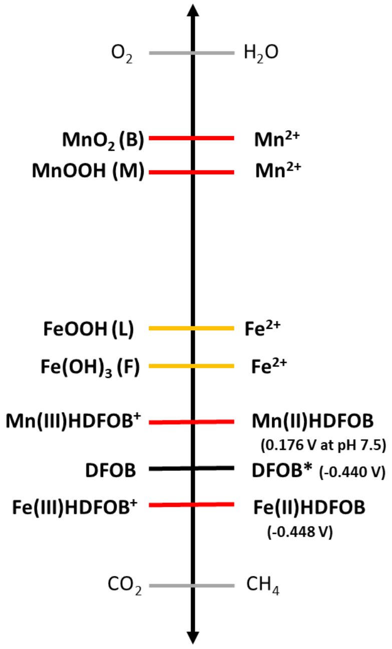

Mn(III)HDFOB/Mn(II)HDFOB[5]

DFOB/DFOB*[6]

Fe(III)HDFOB/Fe(II)HDFOB[7]

Figure S2. XRD patterns of synthesized (a) manganite, (b) lepidocrocite and (c) 2-line ferrihydrite. The mineral sample were dried under ambient air conditions prior to the measurement. Measured using Cu K-alpha radiation (l=1.5406 Å)

(a)

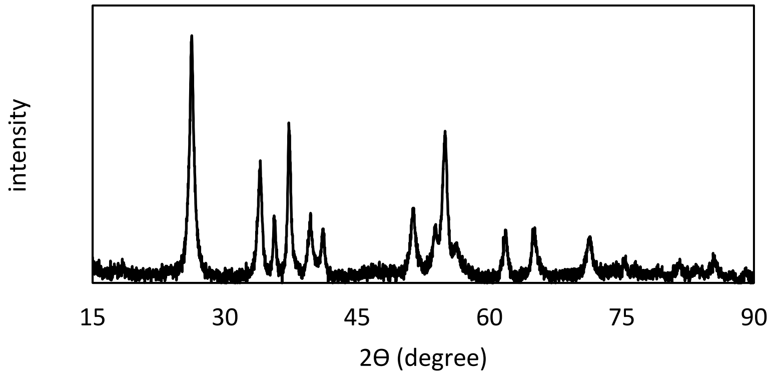

(b)

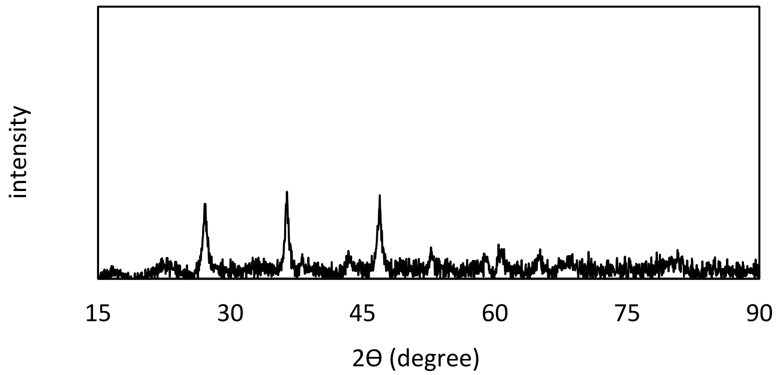

(c)

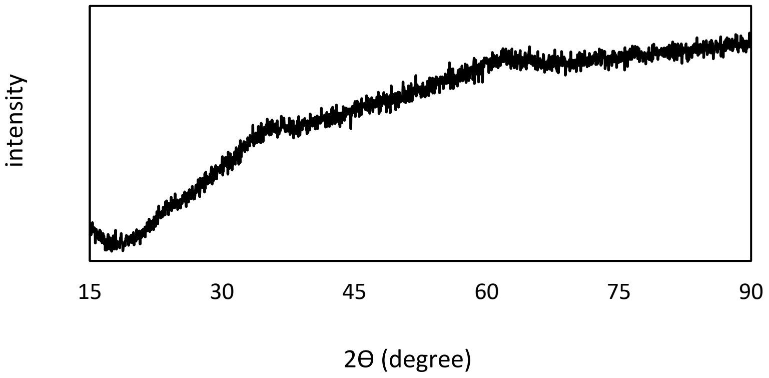

Figure S3. Absorbance of metal-ligand complexes under various conditions.

(a) Fe-DFOB (1:1), pH 7.0, λ_{max}=430 nm

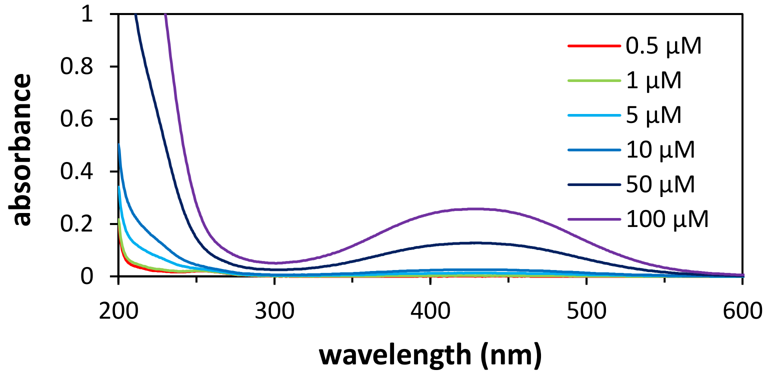

(b) Mn-DFOB (1:1.1), pH 7.0, λ_{max}=310 nm

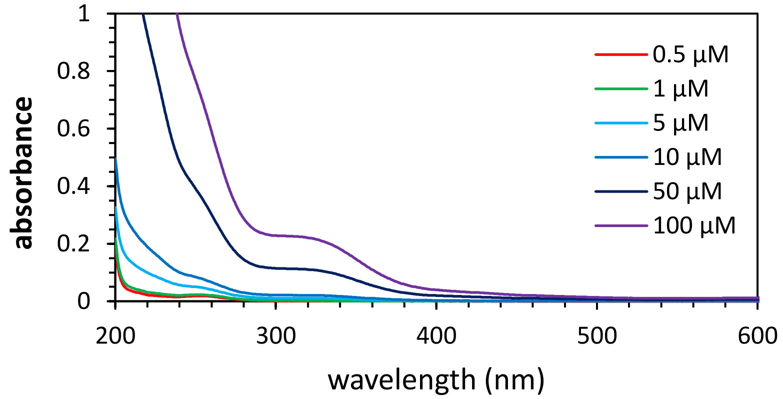

(c) Mn-DFOB (1:1.1) in the presence of 100 µM Mn(II)

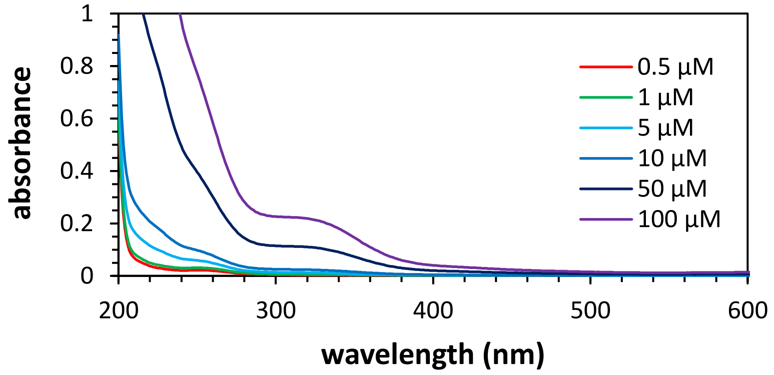

(d) Mn-DFOB (1:1.1) + Fe-DFOB (1:1)

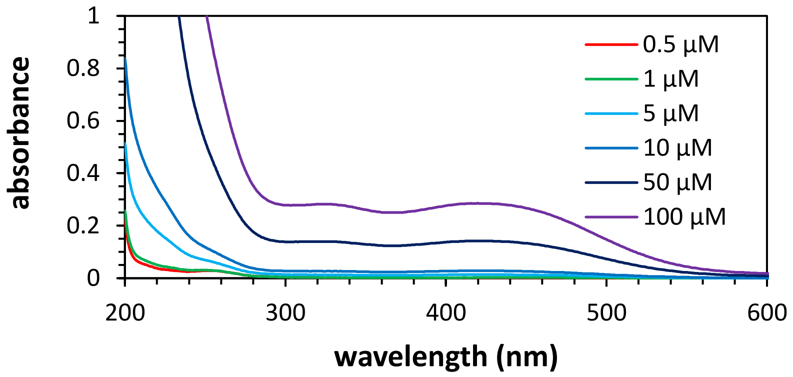

(e) Mn-PP (1:20), pH 8.0, λ_{max}=257 nm

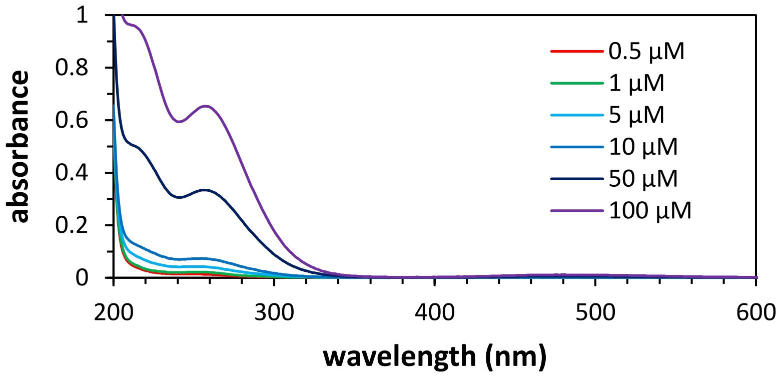

(f) Mn-PP (1:20) in the presence of 100 μM Mn(II) at pH 8.0, λ_{max}=257 nm

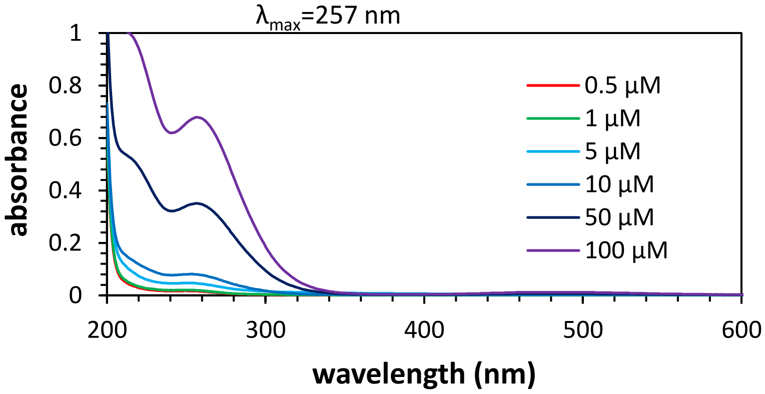

Figure S4. Control experiments: Mn mobilization from manganite (1 mM) and δ-MnO_2 (1 mM) at pH (a) 7.0 and (b) 7.5.

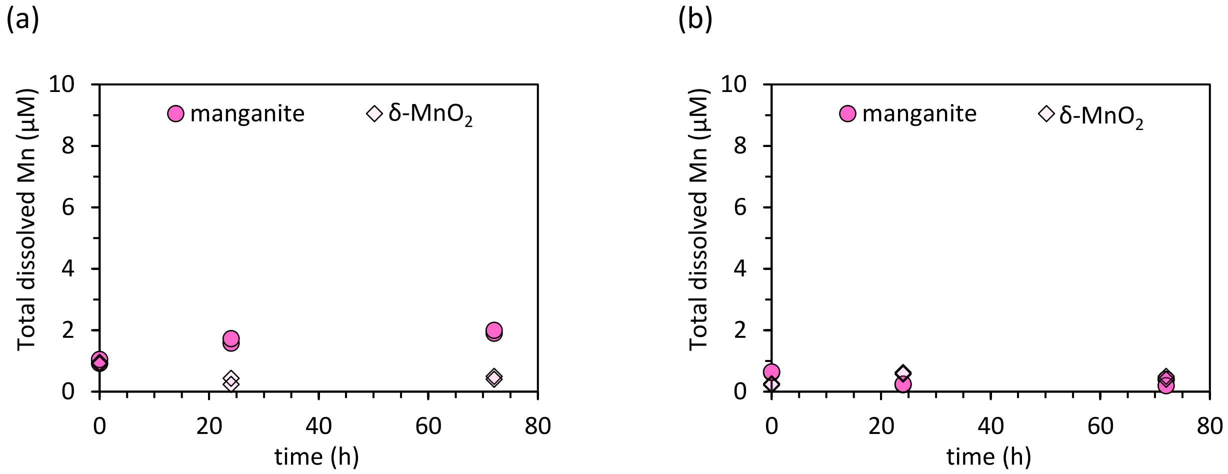

Figure S5. Manganese (Mn) mobilization from manganite (a) and δ-MnO_2 (b) and iron (Fe) mobilization from lepidocrocite (c) and 2-line ferrihydrite (d) by 50 μM DFOB as a function of time at pH 7.5 under oxic conditions in single and mixed mineral suspensions (1 mM Mn and 1 mM Fe, 0.1 M NaCl). Mobilized Mn(III) and Fe(III) are shown as Mn-DFOB (a and b) and Fe-DFOB (c and d), respectively. Complexed DFOB is shown as Mn-DFOB in manganese single mineral systems and the sum of Mn-DFOB and Fe-DFOB in mixed mineral systems (e and f). A different y-axis scale was used for Figure 1b.

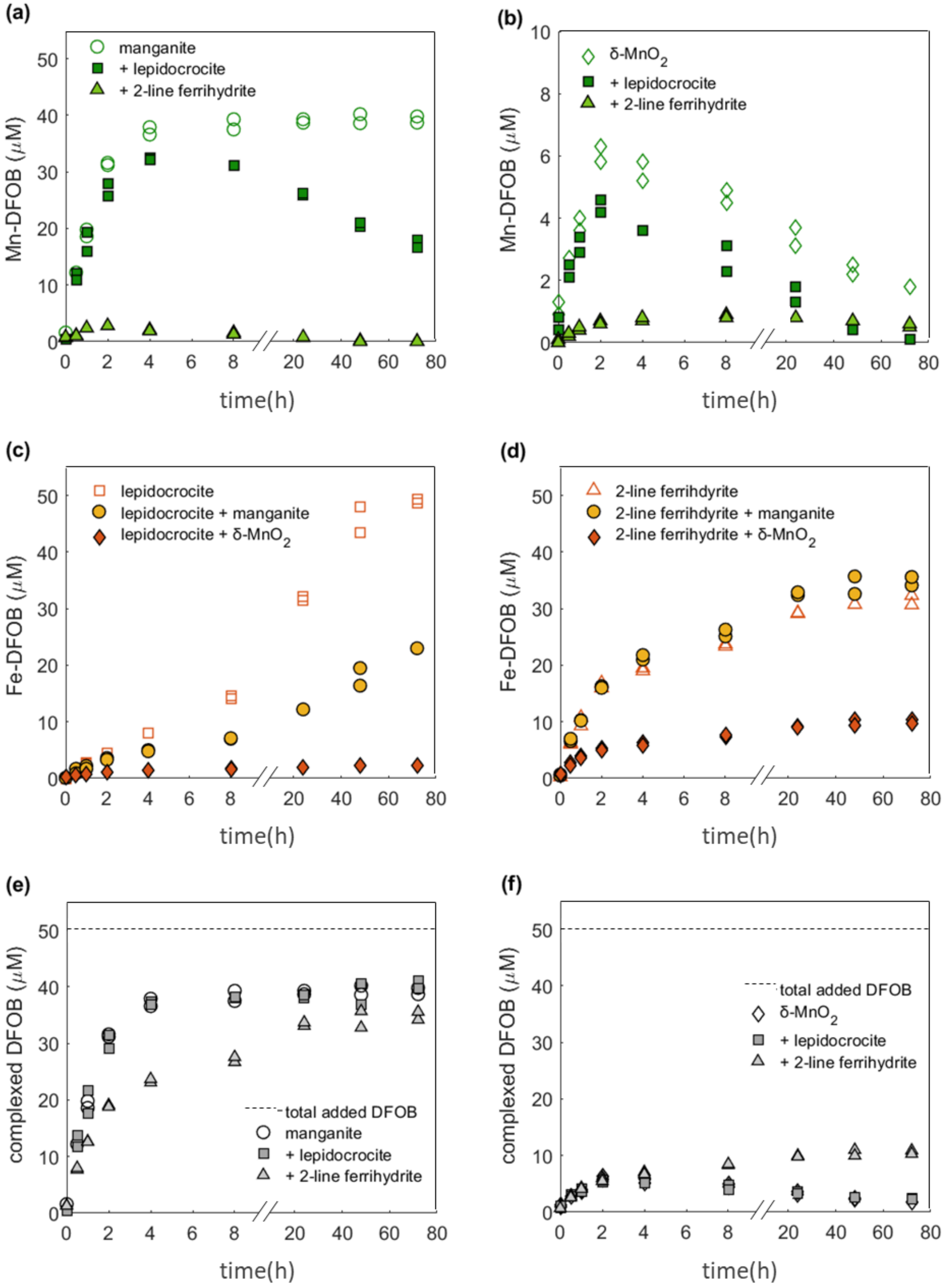

Figure S6. Manganese(II) mobilization by 50 µM DFOB from manganite (a) and δ-MnO2 (b) as a function of time at pH 7.5 under oxic conditions in the presence and absence of lepidocrocite and 2-line ferrihydrite (1 mM Mn, 1 mM Fe, 0.1 M NaCl). Note the difference in the y-axis scale for manganite (a) and δ-MnO2 (b).

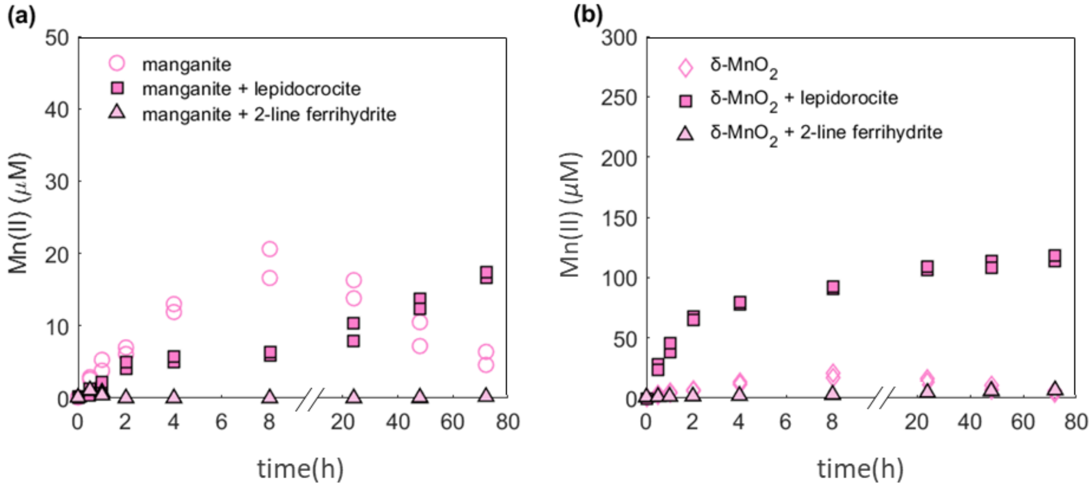

Figure S7. Logarithmic scale of Mn-DFOB, Fe-DFOB and Mn(II) mobilization by 50 µM DFOB from mixed mineral systems (manganite + lepidocrocite (a), manganite + 2-line ferrihydrite (b), δ-MnO_2 + lepidocrocite (c) and δ-MnO_2 + 2-line ferrihydrite (d)).

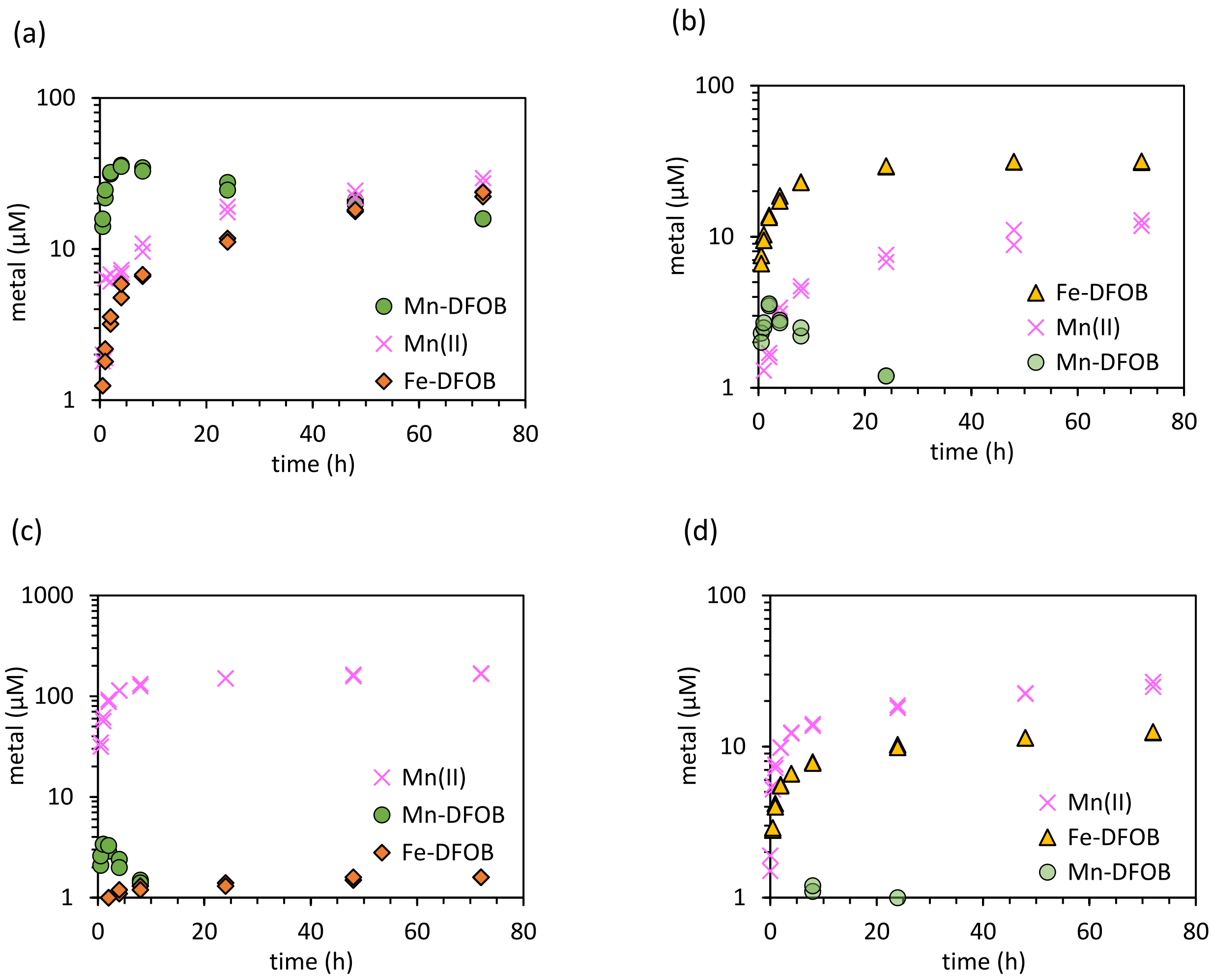

Figure S8. Adsorption isotherms for Mn(II) onto (a) manganite, (b) lepidocrocite and (c) 2-line ferrihydrite (1 mM Mn and 1 mM Fe at pH 7.0 and 7.5, 0.1 M NaCl). Adsorption data was obtained for different Mn(II) concentration ranges: 10 to 500 µM. The equilibration time was 10 minutes. q_{max} and K_D values (Table S6) were calculated based on the Langmuir model and shown here (solid lines).

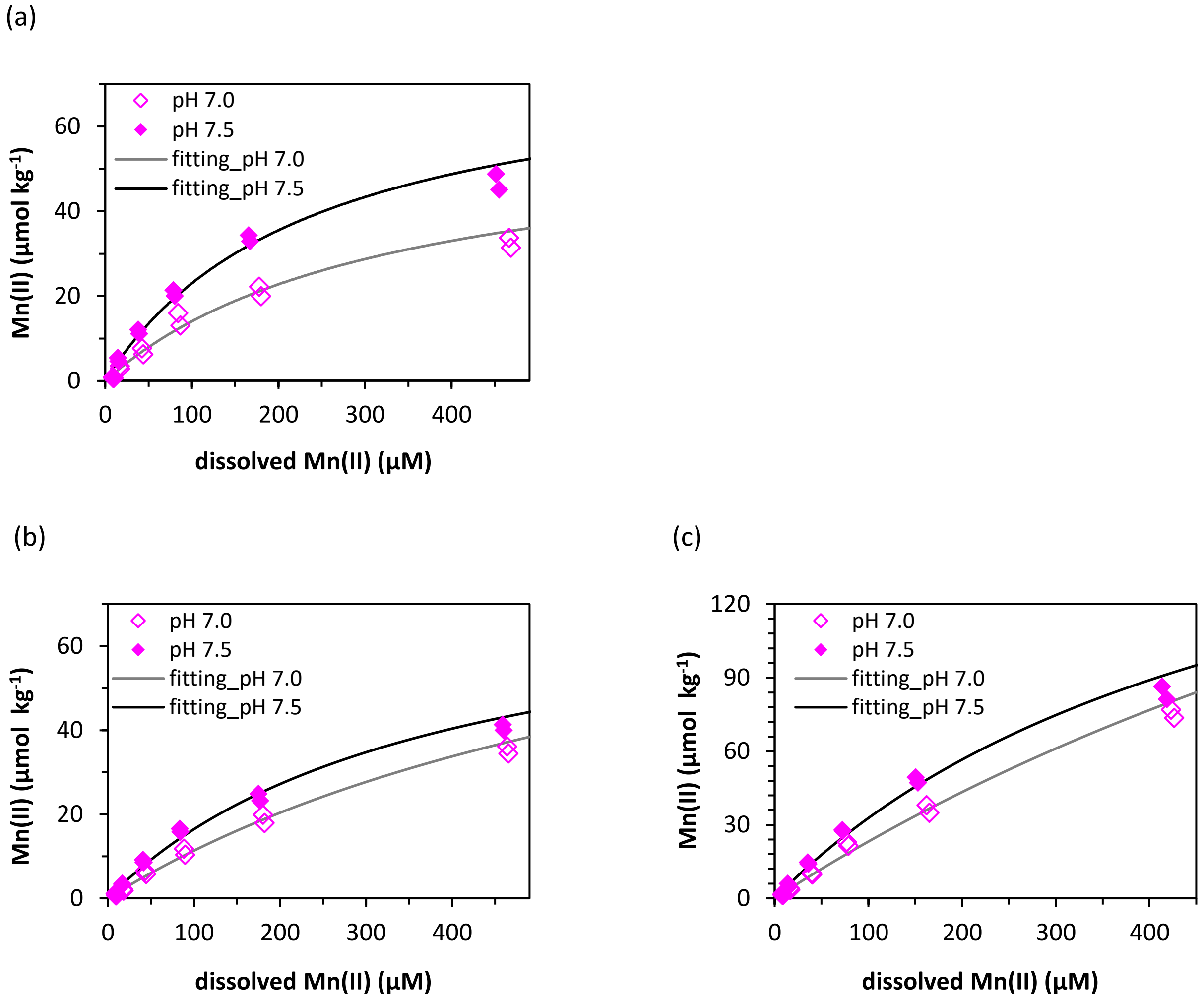

Figure S9. Adsorption isotherms for DFOB onto (a) manganite, (b) δ-MnO_2, (c) lepidocrocite and (d) 2-line ferrihydrite (1 mM Mn and 1 mM Fe at pH 7.0 and 7.5, 0.1 M NaCl). Adsorption data was obtained for different DFOB concentration ranges: 0.5 to 100 µM. The equilibration time was 10 minutes. q_{max} and K_D values (Table S6) were calculated based on the Langmuir model and shown here (solid lines).

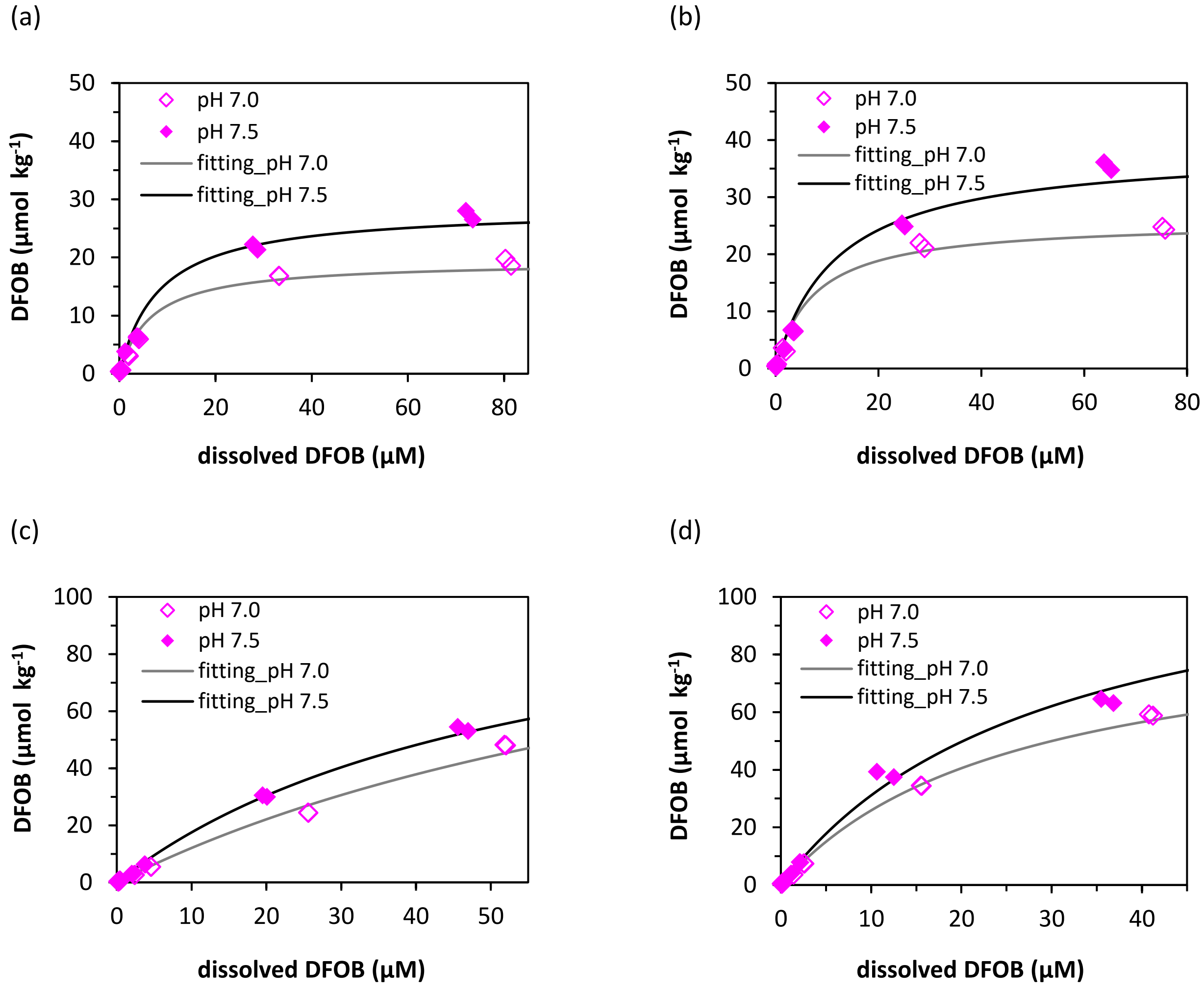

Figure S10. Adsorption isotherms for Mn-DFOB onto (a) manganite, (b) δ-MnO_2, (c) lepidocrocite and (d) 2-line ferrihydrite (1 mM Mn and 1 mM Fe at pH 7.0 and 7.5, 0.1 M NaCl). Adsorption data was obtained for different Mn-DFOB concentration ranges: 0.5 to 100 µM. The equilibration time was 10 minutes. q_{max} and K_D values (Table S6) were calculated based on the Langmuir model and shown here (solid lines).

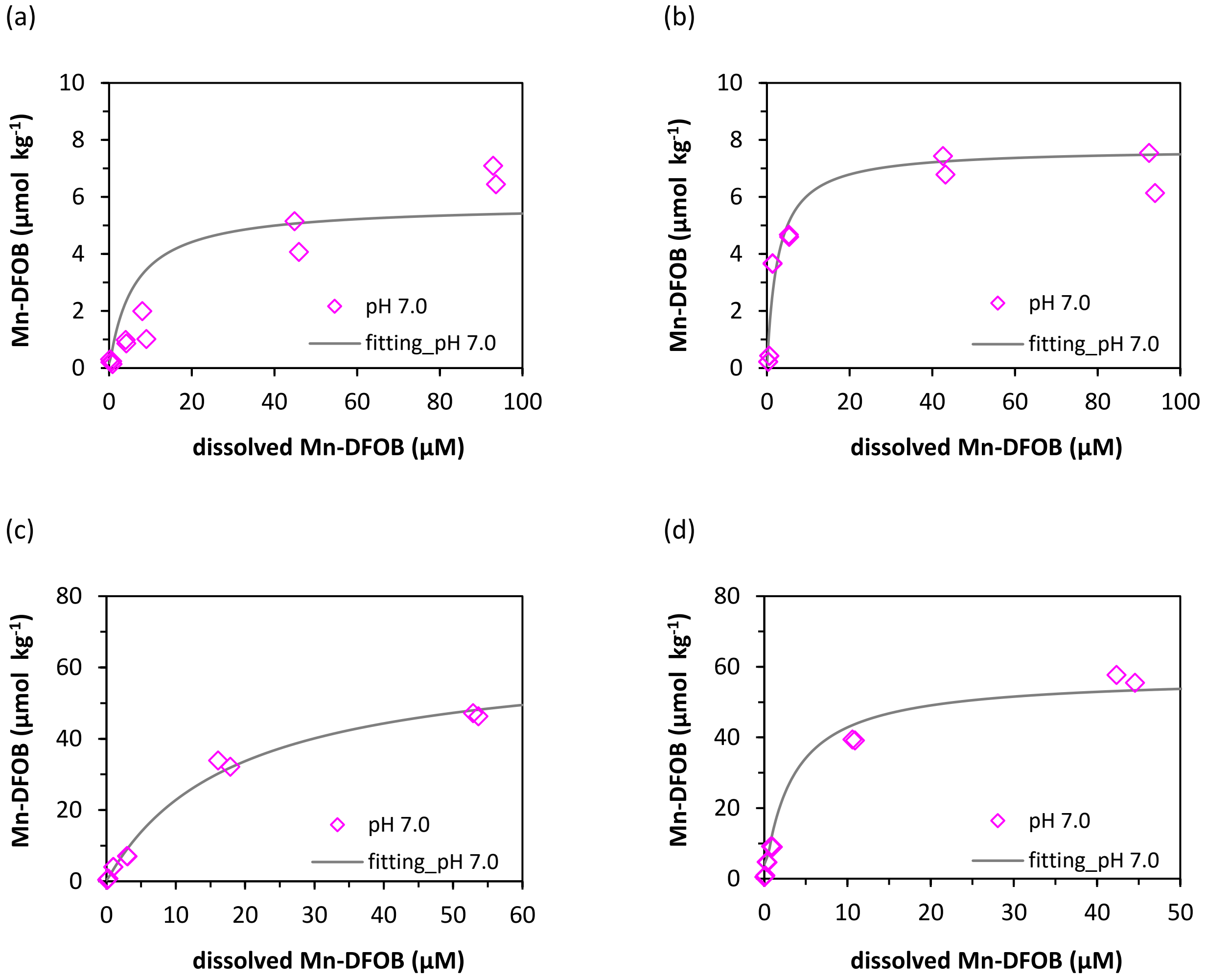

Figure S11. Adsorption isotherms for Fe-DFOB onto (a) manganite, (b) δ-MnO_2, (c) lepidocrocite and (d) 2-line ferrihydrite (1 mM Mn and 1 mM Fe at pH 7.0 and 7.5, 0.1 M NaCl). Adsorption data was obtained for different Fe-DFOB concentration ranges: 0.5 to 100 µM. The equilibration time was 10 minutes. q_{max} and K_D values (Table S6) were calculated based on the Langmuir model and shown here (solid lines).

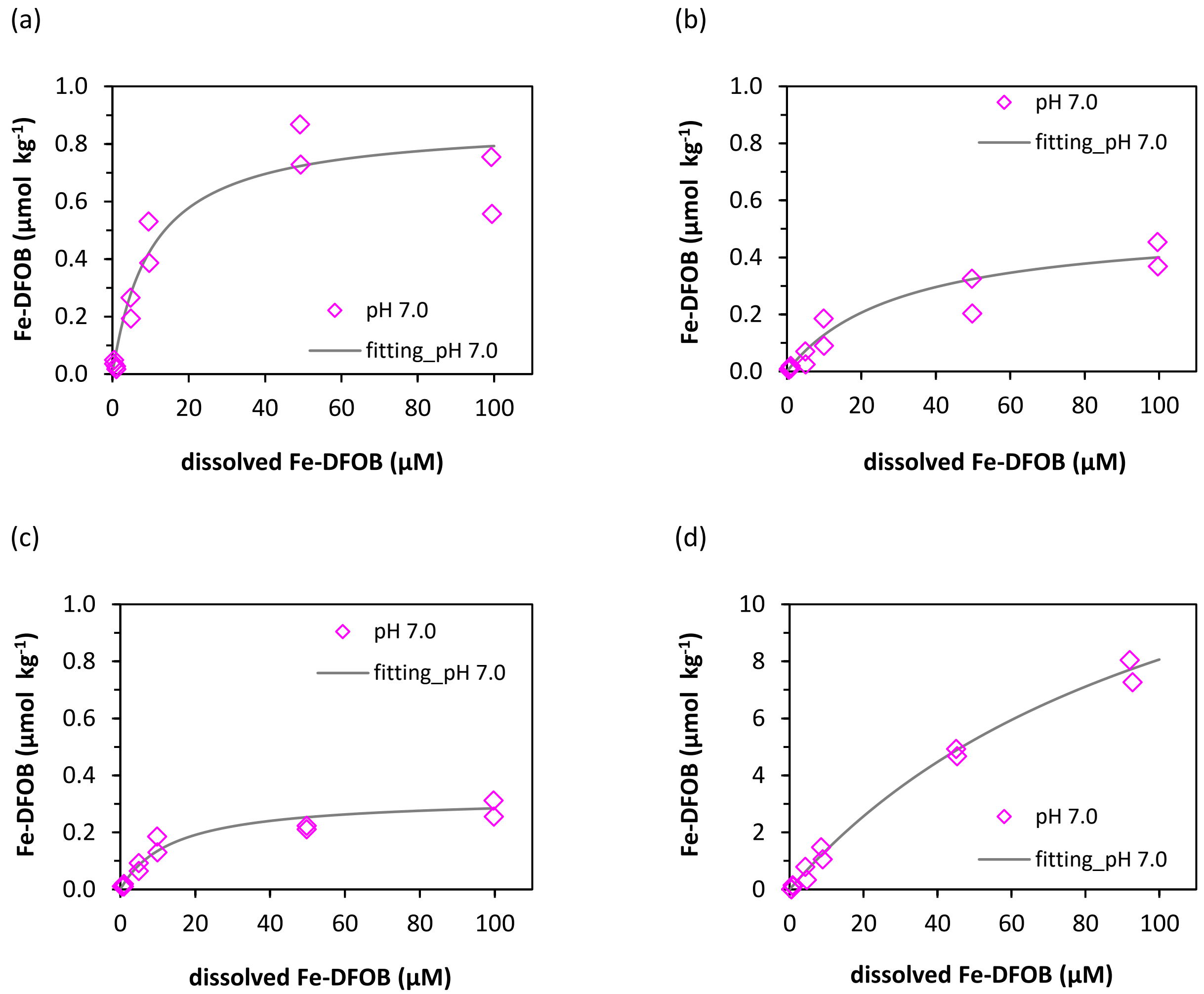

Figure S12. LC-MS result of (a) 50 µM DFOB adjusted to the pH 7.0 and (b) DFOB calibration curve. Mass spectra were recorded in the *m/z* range 100-800 in positive electrospray mode using a Thermo Scientific LTQ-FT ultra instrument. The isotopic pattern of DFOB is in excellent agreement with the theoretically expected pattern, and the mass accuracy is better than 10 ppm. The DFOB calibration curve was calculated based on the intensity. Three parallel masses indicate the isotopic difference in DFOB compound.

(a)

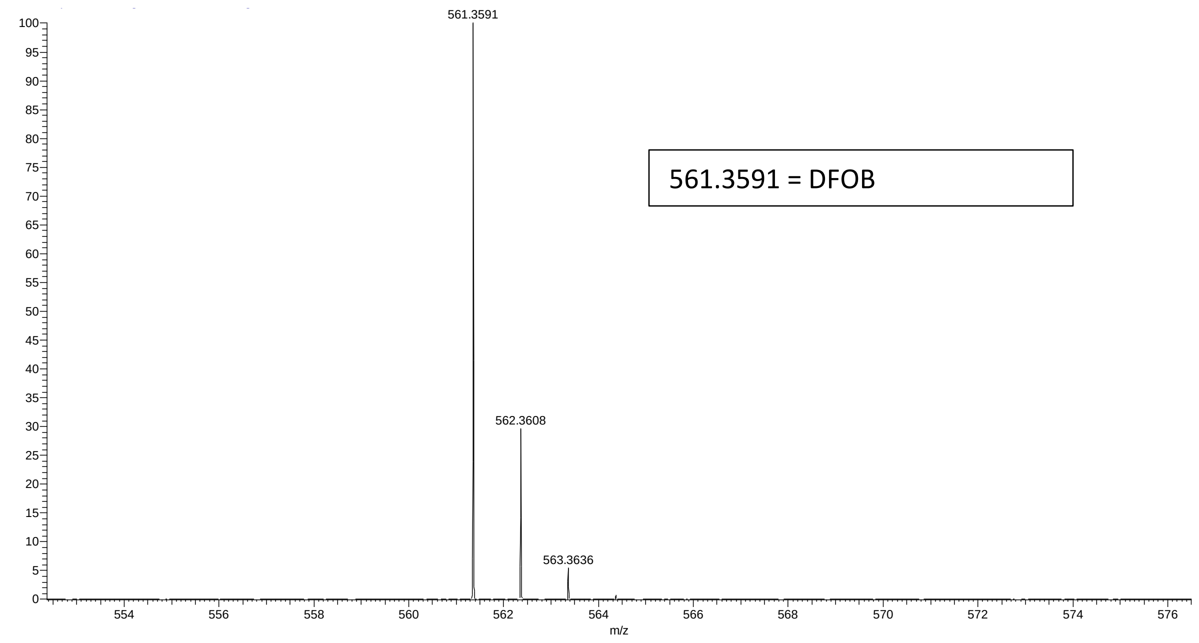

(b)

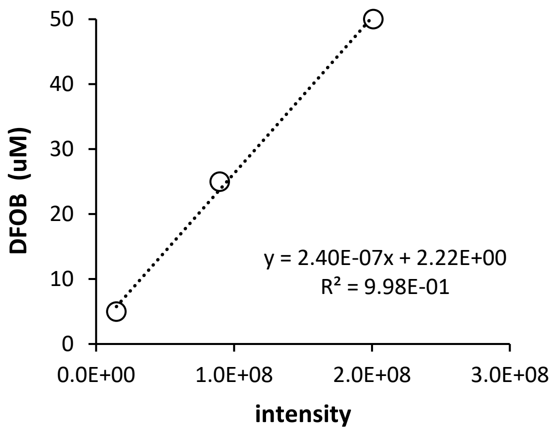

Figure S13. Decomposition products measured upon reaction with 1 mM manganite at pH 7.0 (0 mM NaCl) after 2 h (a) and 72 h (b) of reaction.

(a)

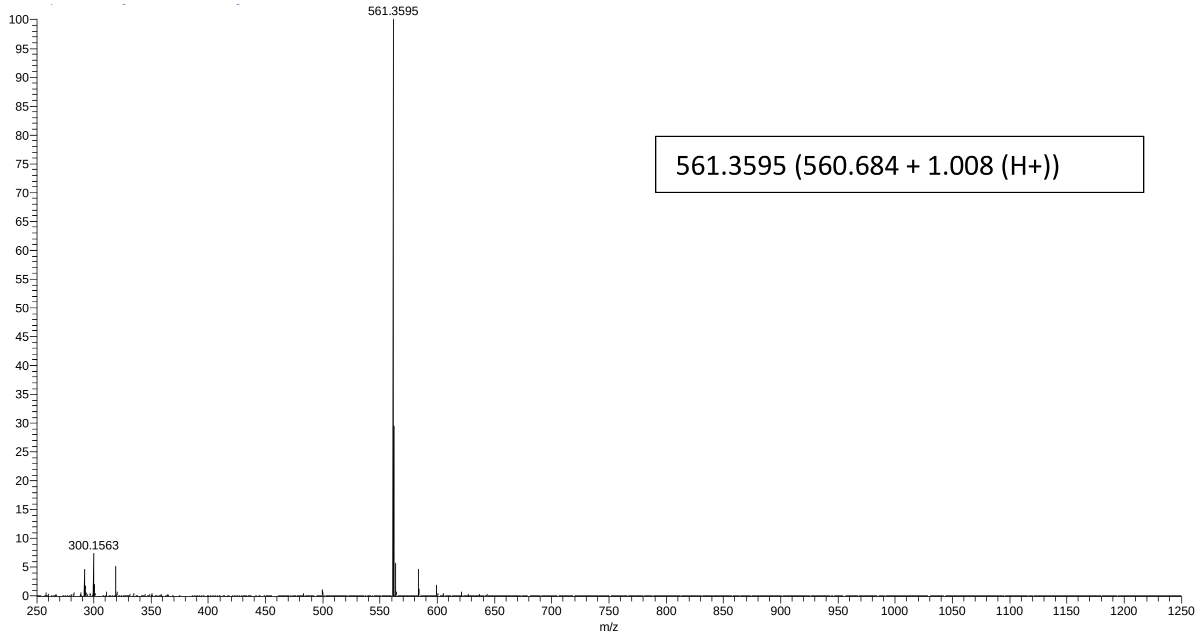

(b)

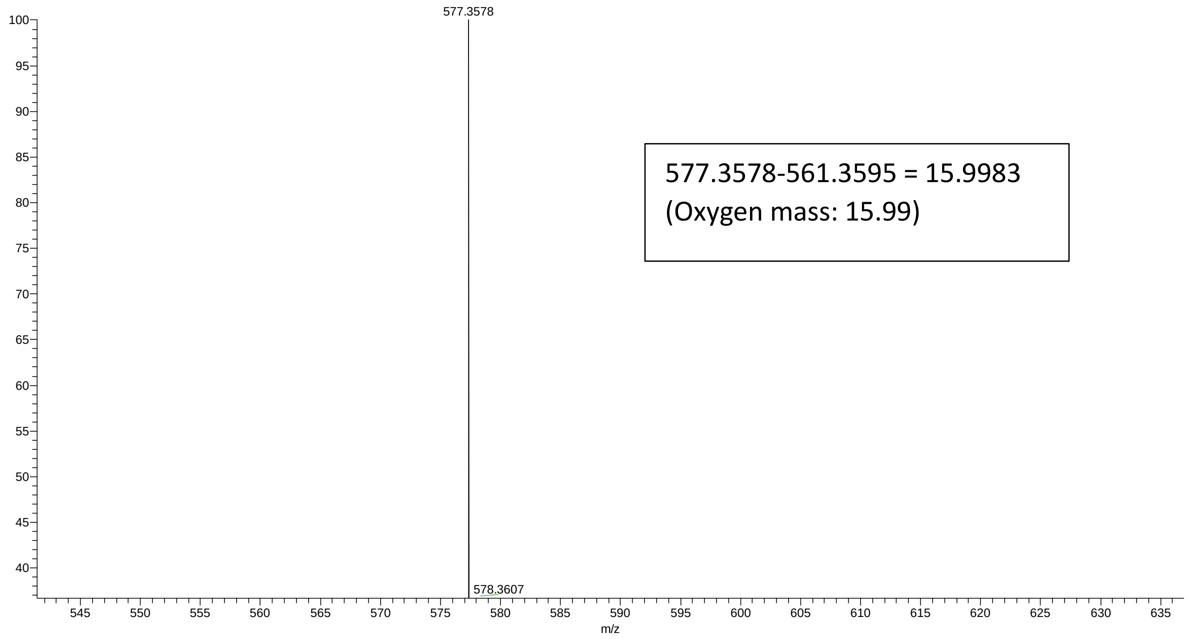

Figure S14. Decomposition products measured upon reaction with 1 mM δ-MnO_2 at pH 7.0 (0 mM NaCl) after 2 h (a, b and c), 24 h (d) and 72 h (e and f) of reaction.

(a) DFOB, 2 h

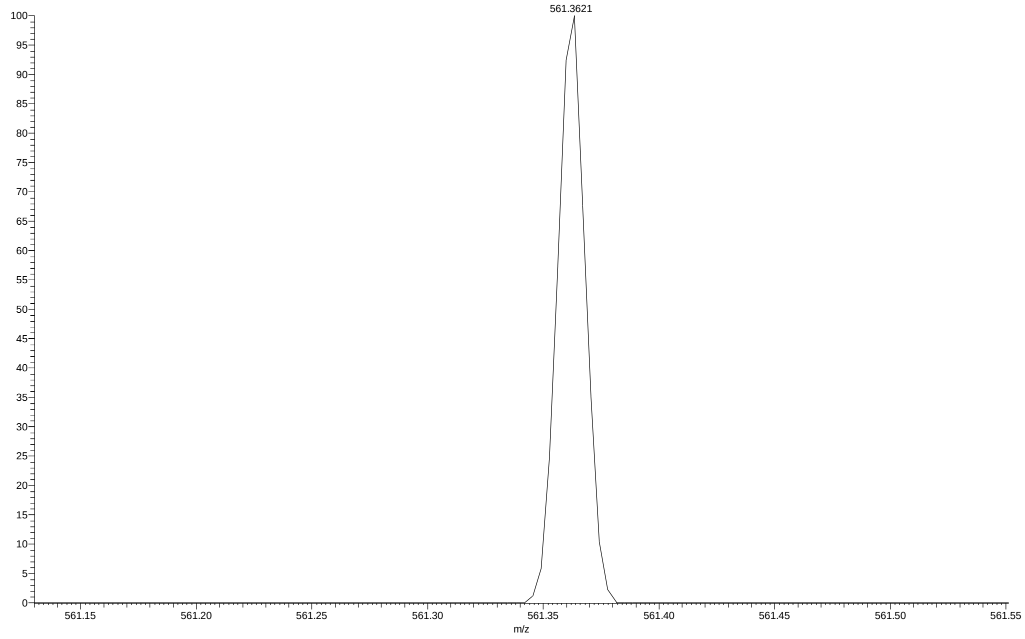

(b) degradation products, 2 h, low mass (less than 100)

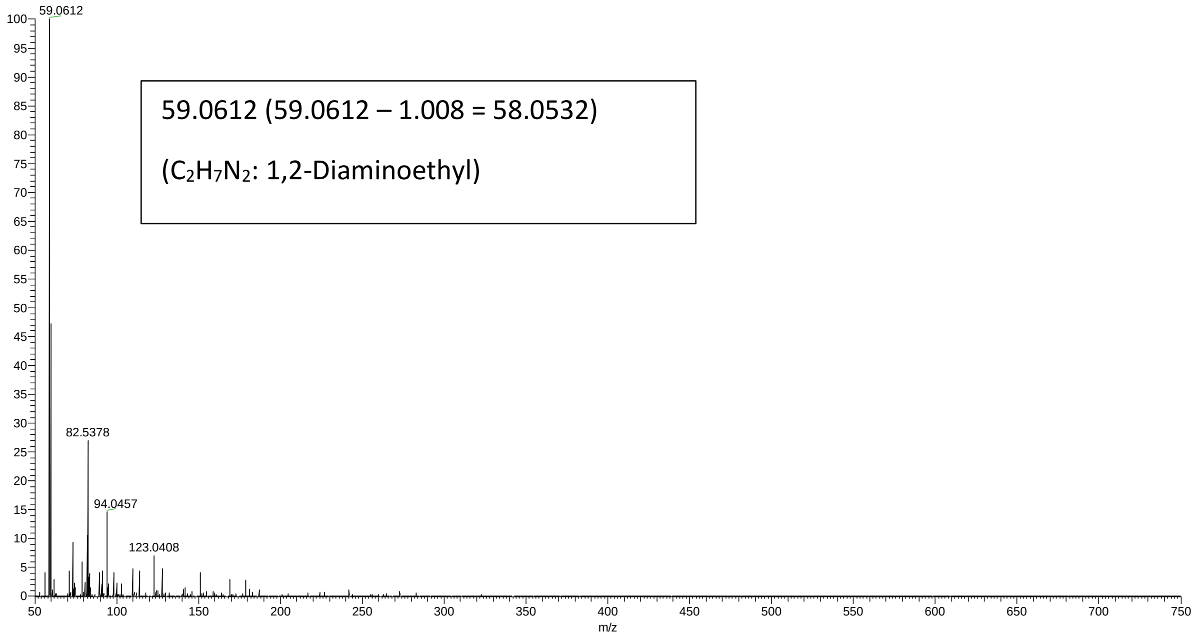

(c) degradation products, 2 h, nagative mode

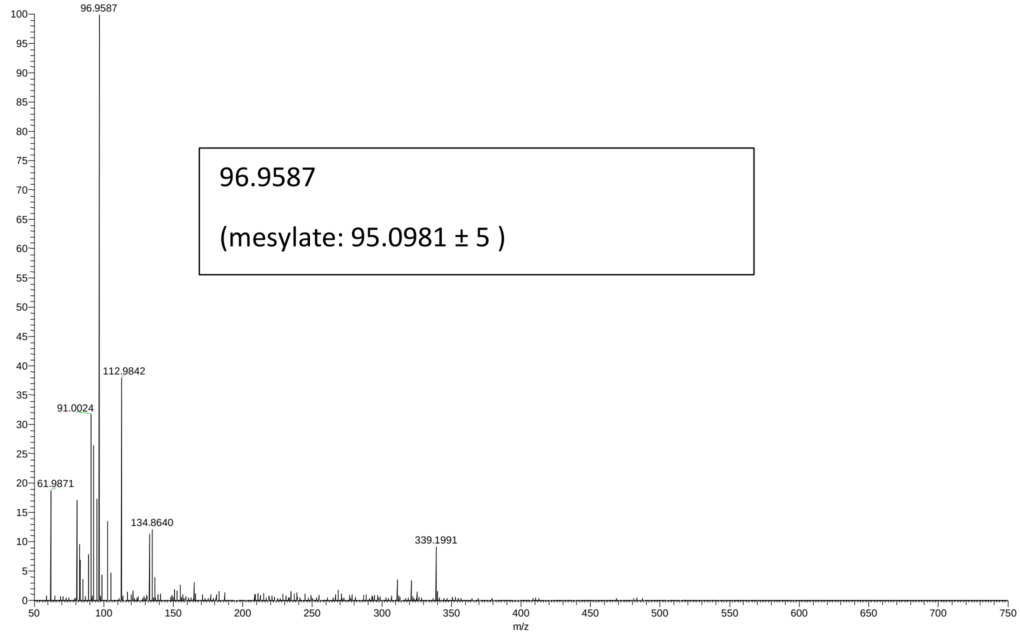

(d) degradation products, 24 h

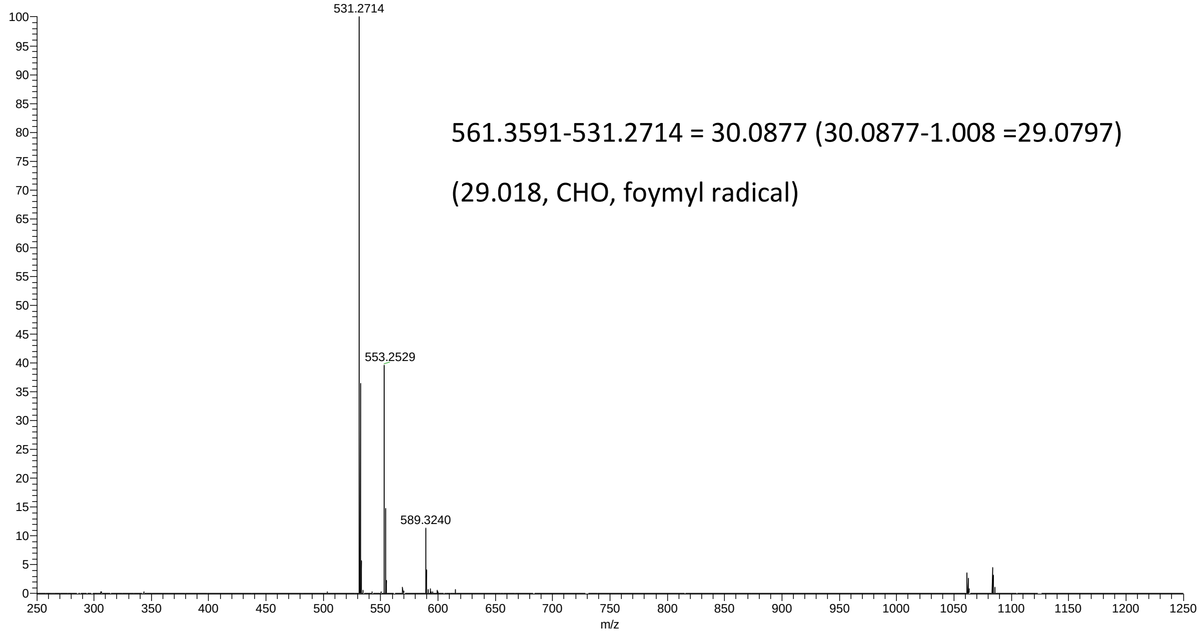

(e) degradation products, 72 h

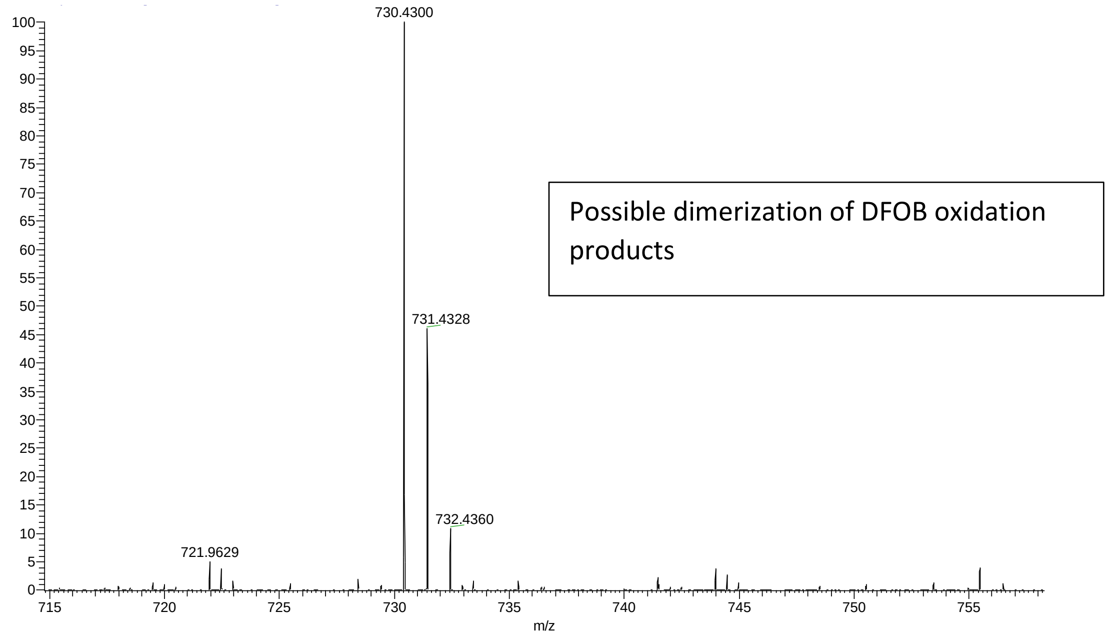

(f) degradation products, 72 h (potential dimer formation)

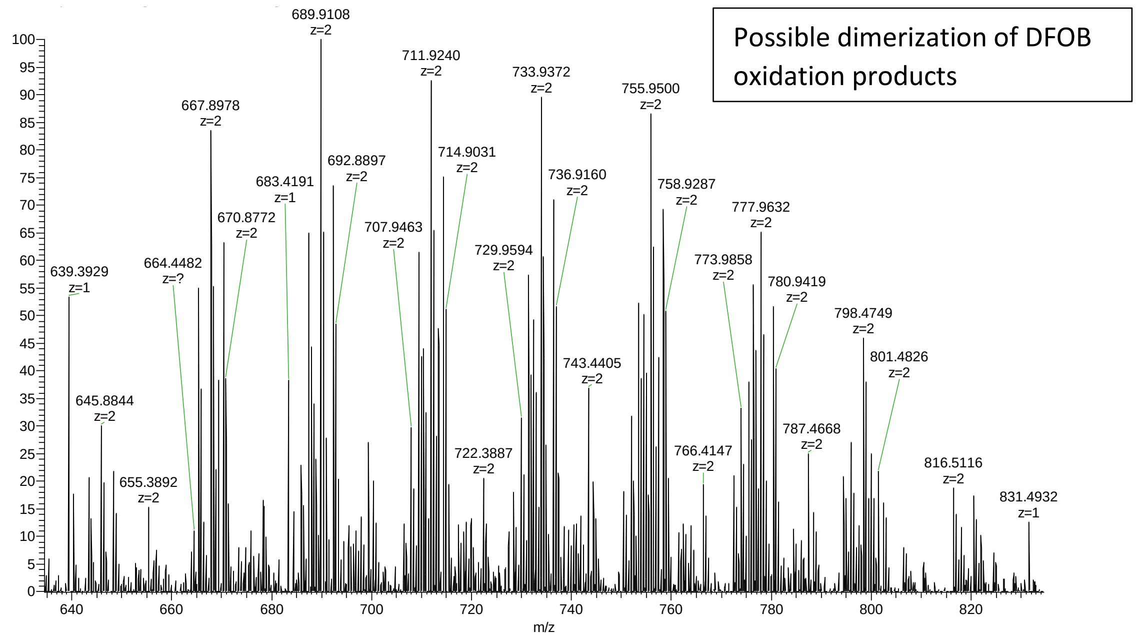

Figure S15. Wet chemistry data for XAS sample type 1: change in Mn and Fe speciation during the reaction between 1 mM Mn-DFOB and 10 mM of Fe as lepidocrocite or 2-line ferrihydrite (0.1 M NaCl) as a function of time at pH 7.0 under oxic conditions. Changes in the concentration of the Mn species are shown in (a) as Mn-DFOB, (c) as Mn(II), and as total dissolved Mn (d). Fe mobilized as Fe-DFOB is shown in (b). Complexed DFOB species are shown in (e).

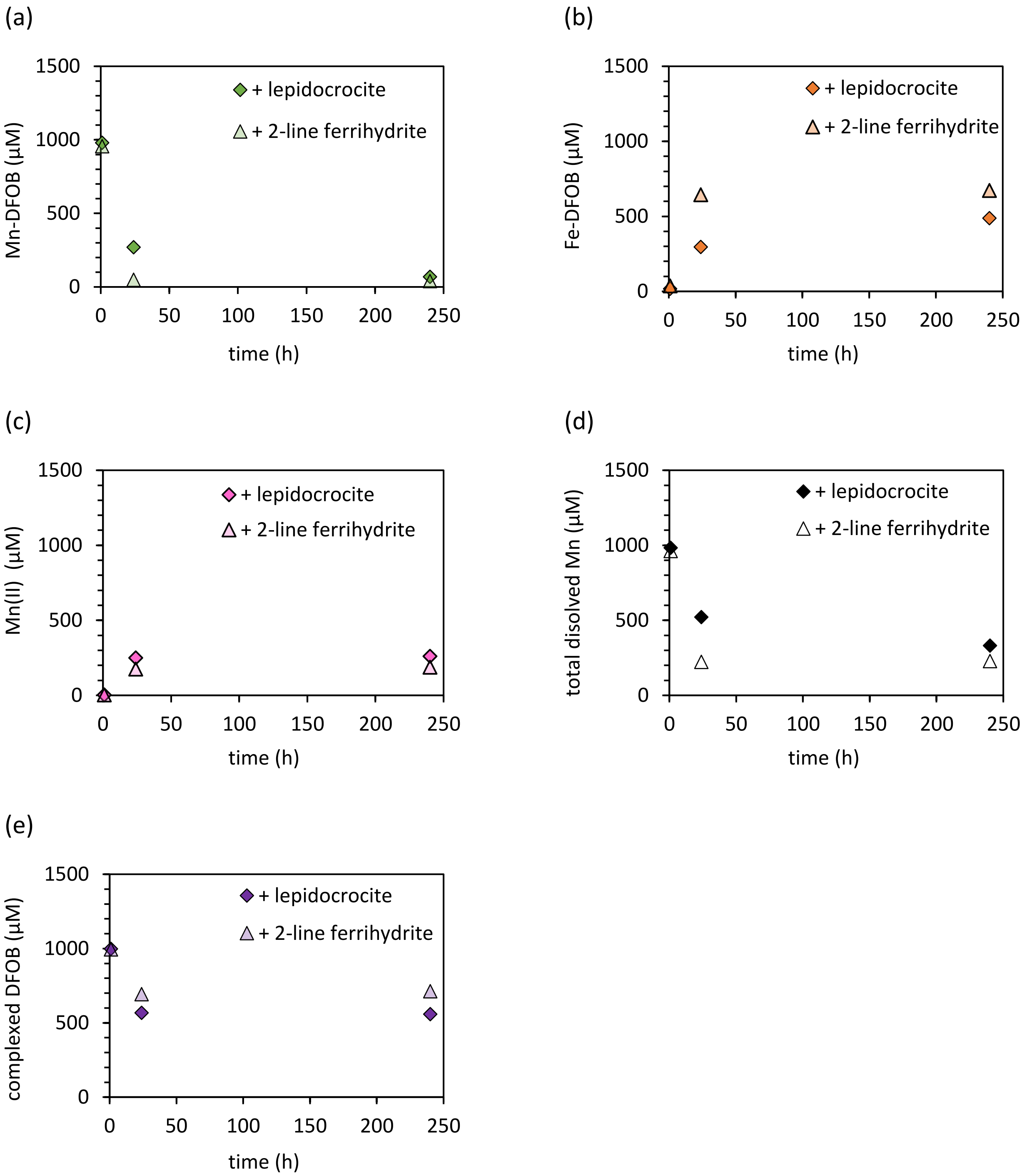

Figure S16. Wet chemistry data for XAS sample type 2: Mn and Fe mobilization from manganite (a, c and e) and δ-MnO_2 (b, d and f) in the presence and absence of lepidocrocite and 2-line ferrihydrite (10 mM Mn, 10 mM Fe 0.1 M NaCl) by 1 mM DFOB as a function of time at pH 7.0 under oxic conditions. Mn mobilized is shown in as Mn-DFOB (a and b), and as Mn(II) (e and f). Fe mobilized is shown in as Fe-DFOB (c and d). This supplementary experiment has been conducted normal batch system.

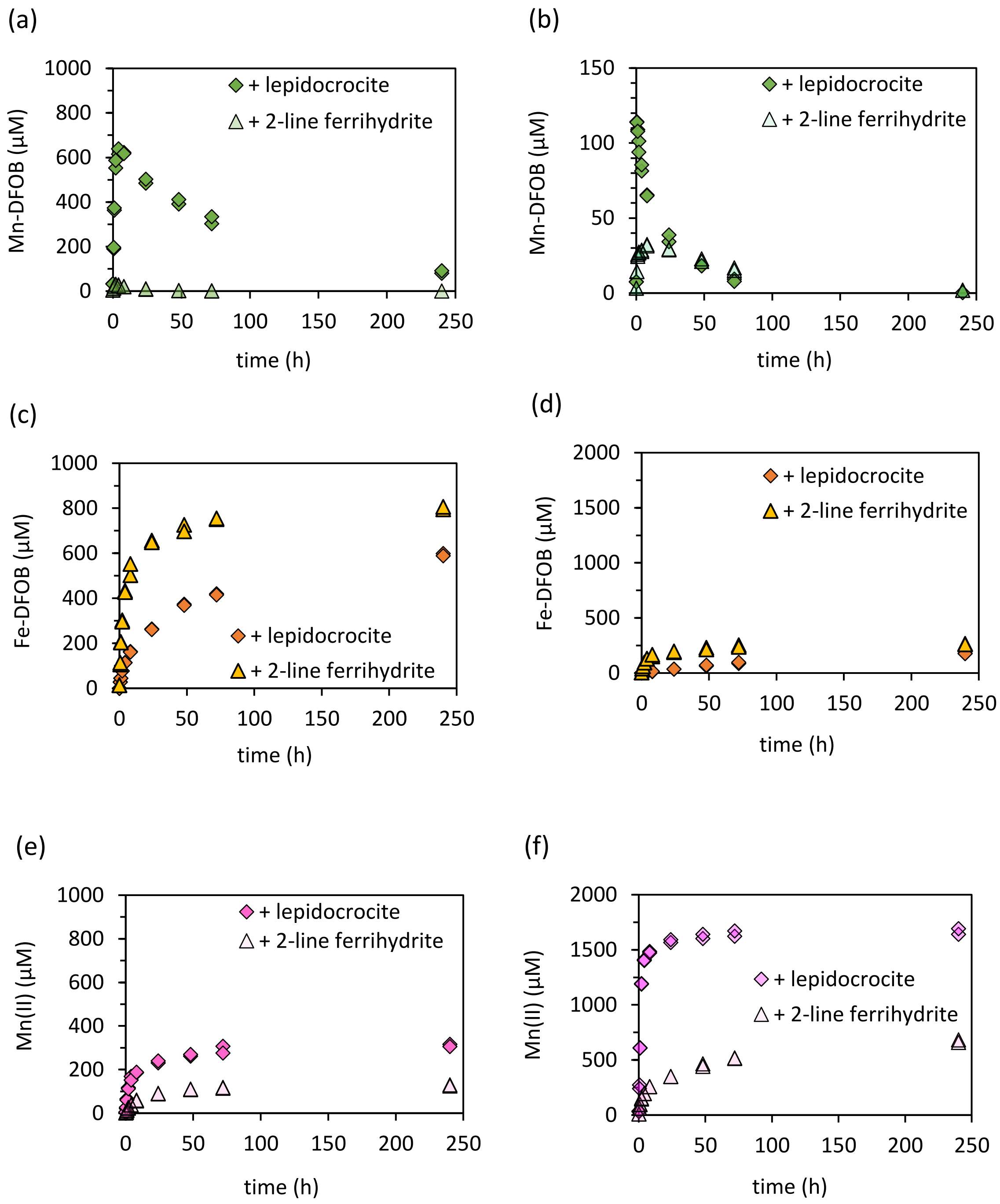

Figure S17. Mn K-edge EXAFS spectra (a) and Fourier transform of the Mn K-edge EXAFS (b) (k 3 χ(k)) spectra (k = 2–8 $Å^{-1}$) of reference samples ($MnSO_4$, bixbyite, groutite, manganite and δ-MnO_2) and experimental samples (1 mM Mn-DFOB added to 10 mM lepidocrocite and 10 mM 2-line ferrihydrite, and 1 mM DFOB added to 10 mM of lepidocrocite and 2-line ferrihydrite in the presence of manganite and δ-MnO_2). Linear combination fit results are shown by the dashed lines.

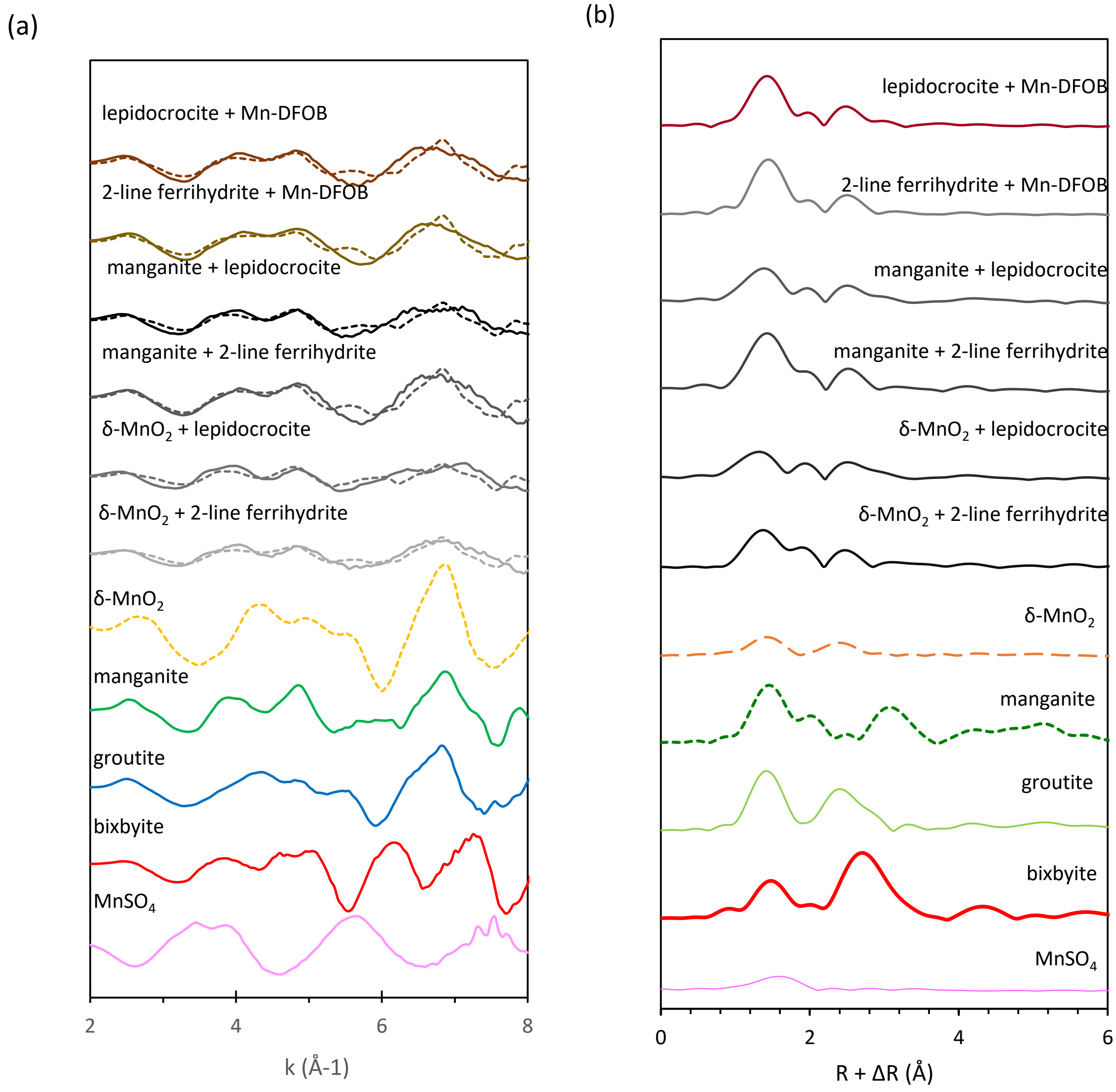

Text S1. Mn-DFOB (λ_{max}=310 nm) and Fe-DFOB (λ_{max}=430 nm) concentration calculation.

The spectral interference of Fe-DFOB complexes inhibit a direct quantification of Mn-DFOB concentration. The absorbance and the extent of interference of Fe-DFOB increases linearly with the Fe-DFOB concentration. Hence the absorbance at 310 nm in solutions containing Fe-DFOB and Mn-DFOB can be described with:

$$abs_{310} = \varepsilon_{Mn\text{-}DFOB}\, l\ C_{Mn\text{-}DFOB} + \varepsilon_{Fe\text{-}DFOB\text{-}interference}\, l\ C_{Fe\text{-}DFOB} \qquad (1)$$

$$abs_{430} = \varepsilon_{Fe\text{-}DFOB}\, l\ C_{Fe\text{-}DFOB} + \varepsilon_{Mn\text{-}DFOB\text{-}interference}\, l\ C_{Mn\text{-}DFOB} \qquad (2)$$

$$C_{Fe(tot)} = C_{Fe-DFOB} \qquad (3)$$

$$C_{Mn(tot)} = (C_{Mn(II)}) + C_{Mn-DFOB} \qquad (4)$$

Where abs310 is the measured absorbance at 310 nm, $\varepsilon_{Mn\text{-}DFOB}$ and $\varepsilon_{Fe\text{-}DFOB}$ are the extinction coefficients for Mn-DFOB and Fe-DFOB, respectively. $\varepsilon_{Mn\text{-}DFOB\text{-}interference}$ and $\varepsilon_{Fe\text{-}DFOB\text{-}\ interference}$ are the extinction coefficients for Mn-DFOB interference at wavelength 430 nm and Fe-DFOB interference at wavelength 310 nm, respectively. *l* is the optic path length (1 cm) and $C_{Mn\text{-}DFOB}$ and $C_{Fe\text{-}DFOB}$ are the Mn-DFOB and Fe-DFOB concentration, respectively. The total Fe solution concentration measured by ICP-MS equals to $C_{Fe\text{-}DFOB}$. The total Mn solution concentration measured by ICP-MS equals the sum of the Mn-DFOB concentrations. Mn(II) does not interfere with absorbance at 310 and 430 nm. In the calculation as well as the solution containing Mn-DFOB and Fe-DFOB do not contain Mn(II). Combining equation 1, 2, 3 and 4 provides the following expression for the Mn-DFOB concentration:

$$\boldsymbol{C_{Mn-DFOB}} = \frac{\boldsymbol{abs_{310}} - (\boldsymbol{\varepsilon_{Fe-DFOB-interference}} \times \boldsymbol{l} \times \boldsymbol{C_{Fe-DFOB}})}{\boldsymbol{l} \times \boldsymbol{\varepsilon_{Mn-DFOB}}} \qquad (5)$$

References

1. Murray JW. Surface chemistry of hydrous manganese-dioxide *Journal of Colloid and Interface Science.* 1974;46(3):357-371.

2. Marafatto FF, Lanson B, Pena J. Crystal growth and aggregation in suspensions of delta-MnO2 nanoparticles: implications for surface reactivity. *Environmental Science-Nano.* Feb 2018;5(2):497-508.

3. Ramstedt M, Andersson BM, Shchukarev A, Sjoberg S. Surface properties of hydrous manganite (gamma-MnOOH). A potentiometric, electroacoustic, and X-ray photoelectron spectroscopy study. *Langmuir.* Sep 2004;20(19):8224-8229.

4. Kosmulski M. The pH dependent surface charging and points of zero charge. VIII. Update. *Advances in Colloid and Interface Science.* Jan 2020;275.

5. Duckworth OW, Sposito G. Siderophore-manganese(III) interactions. I. Air-oxidation of manganese(II) promoted by desferrioxamine B. *Environmental Science & Technology.* Aug 2005;39(16):6037-6044.

6. Cooper SR, McArdle JV, Raymond KN. Siderophore electrochemistry-relation to intracellular iron release mechanism. *Proceedings of the National Academy of Sciences of the United States of America.* 1978;75(8):3551-3554.

7. Boukhalfa H, Reilly SD, Neu MP. Complexation of Pu(IV) with the natural siderophore desferrioxamine B and the redox properties of Pu(IV)(siderophore) complexes. *Inorganic Chemistry.* Feb 2007;46(3):1018-1026.
